# A flagellin-conjugate protein induces dual NLRC4- and NLRP3-inflammasome activation which modulates inflammatory cytokine secretion from macrophages

**DOI:** 10.3389/fimmu.2023.1136669

**Published:** 2023-03-21

**Authors:** Yen-Ju Lin, Annette Jamin, Sonja Wolfheimer, Anna Fiedler, Ann-Christine Junker, Alexandra Goretzki, Stephan Scheurer, Stefan Schülke

**Affiliations:** Molecular Allergology, Paul-Ehrlich-Institut, Langen, Germany

**Keywords:** flagellin fusion protein, inflammasome, NLRP3, NLRC4, macrophage

## Abstract

**Background:**

A recombinant fusion protein combining the adjuvant and TLR5-ligand flagellin with the major birch pollen allergen Bet v 1 (rFlaA:Betv1) has been suggested to prevent the manifestation of birch allergy. Noteworthy, rFlaA:Betv1 induced both pro- and anti-inflammatory responses which were differentially regulated. However, the mechanism by which flagellin fusion proteins modulate allergen-specific immune responses, especially the mechanisms underlying IL-1β secretion and their contribution to the overall immune responses remains elusive.

**Objective:**

To investigate the mechanisms underlying the production of IL-1β from rFlaA:Betv1 stimulated macrophages.

**Methods:**

Macrophages were derived from mouse peritoneal-, human buffy-coat-, and PMA-differentiated THP-1 (wild type or lacking either ASC, NLRP3, or NLRC4) cells. Macrophages were stimulated with non-modified rFlaA:Betv1, mutant variants lacking either the flagellin DC0 domain or a sequence motif formerly described to mediate TLR5-activation, and respective controls in the presence or absence of inhibitors interfering with MAPK- and NF*κ*B-signaling. Cytokine secretion was analyzed by ELISA and intracellular signaling by Western Blot. To study the contribution of IL-1β to the overall immune responses, IL1R-deficient mouse peritoneal macrophages were used.

**Results:**

rFlaA:Betv1 consistently activated all types of investigated macrophages, inducing higher IL-1β secretion compared with the equimolar mixture of both proteins. rFlaA:Betv1-induced activation of THP-1 macrophages was shown to be independent of either the TLR5-activating sequence motif or the flagellin DC0 domain but depended on both NLRP3- and NLRC4-inflammasomes. In addition, NFκB and SAP/JNK MAP kinases regulated rFlaA:Betv1-induced inflammasome activation and cytokine secretion by modulating pro-Caspase-1- and pro-IL-1β-expression in THP-1 macrophages. Finally, lack of IL-1β positive feedback *via* the IL1R strongly diminished the rFlaA:Betv1-induced secretion of IL-1β, IL-6, and TNF-α from peritoneal macrophages.

**Conclusion:**

The mechanisms contributing to rFlaA:Betv1-induced IL-1β secretion from macrophages were shown to be complex, involving both NLRC4- and NLRP3-inflammsomes, as well as NFκB- and SAP/JNK MAP kinase-signaling. Better understanding the mechanisms regulating the activation of immune cells by novel therapeutic candidates like the rFlaA:Betv1 fusion protein will allow us to further improve and develop new treatment strategies when using flagellin as an adjuvant.

## 1 Introduction

Identification of novel adjuvants is an important topic for improving both allergen-specific immunotherapy and vaccine development. Among the types of adjuvants studied so far, ligands that activate pattern recognition receptors (PRRs) have an intrinsic immune activation potential, making them attractive adjuvant candidates for vaccine development. Bacterial flagellin, also known as “Toll”-like receptor 5 (TLR5)-ligand (1), has shown its potential to modulate immune responses as a novel adjuvant (2). However, the underlying mechanisms are not fully understood. In an effort to test the potential of flagellin as a novel adjuvant, we recently generated a recombinant fusion protein, rFlaA:Betv1, that combines the TLR5-ligand flagellin A from *Listeria monocytogenes* with the major birch pollen allergen Bet v 1 (3). Such flagellin-containing fusion proteins efficiently target the fused antigen to TLR5-expressing target cells *in vivo*, which, in case of antigen presenting cells (APCs), were shown to take up, process, and present the fused antigen in the context of the flagellin-mediated immune cell activation, resulting in superior induction of immune responses (4–7). In line with this, flagellin-containing fusion proteins incorporating influenza antigens were shown to efficiently induce immune responses in elderly patients with a good safety profile (8–13).

In our previous studies, rFlaA:Betv1 efficiently suppressed sensitization towards Bet v 1 *in vivo* (3) while also activating mouse bone marrow-derived myeloid dendritic cells (mDCs) (3, 7, 14), bone marrow-derived macrophages (BMDMs) (5), lung epithelial cells (15), and naïve mouse and human B cells (6) more strongly than the equimolar mixture of both proteins either provided alone or as a non-fused mixture. Here, rFlaA:Betv1 induced a pro-inflammatory cytokine production and the subsequent formation of Bet v 1-specific Th1 responses with Th2-suppressing capacity while also mediating a prominent production of the anti-inflammatory cytokine IL-10 in all investigated types of APCs (mDCs (3, 7, 14), BMDMs (7), and B cells (8)), which was shown to significantly suppress Th2 responses.

More detailed mechanistic analyses also showed the anti-inflammatory IL-10 secretion induced by rFlaA:Betv1 in mDCs and BMDMs to be dependent on mechanistic target of rapamycin (mTOR), a master regulator of both cellular metabolism and immune function (16). In contrast, rFlaA:Betv1-induced pro-inflammatory cytokine secretion in mDCs and BMDM was shown to be largely mTOR-independent but depended on MAP kinase signaling (5, 14).

While the mechanisms contributing to the secretion of the anti-inflammatory cytokine IL-10 and some pro-inflammatory cytokines (e.g. IL-6 and TNF-α) by rFlaA:Betv1-stimulated mouse immune cells are meanwhile well investigated, the mechanisms underlying the prominent production of IL-1β from different rFlaA:Betv1-stimulated cell types, especially in the human system, remained elusive.

IL-1β is a highly potent, pyrogenic cytokine whose production needs to be tightly regulated in order to prevent unwanted tissue damage (17). Therefore, the canonical production of bio-active IL-1β is a two-step process. In the first step, the recognition of pathogen-associated molecular patterns (PAMPs) by PRRs such as TLRs or TNF-α and IL-1-family cytokines binding to the receptors TNFR and IL1R1, results in the transduction of an activating signal into the nucleus of the respective cells, which promote, among other things, the production of the inactive IL-1β pro-form by immune cells (18, 19). In a second step, the intracellular recognition of PAMPs like the components of the type III secretion system or flagellin *via* NLR Family CARD Domain Containing 4 (NLRC4) (19), or various PAMPs and danger-associated molecular patterns (DAMPs) by NOD-, LRR- and pyrin domain-containing protein 3 (NLRP3) receptors (18) results in the formation of the inflammasome complex. Inflammasomes are large, high-molecular, wheel-shaped complexes consisting of NLRs, the adaptor protein apoptosis-associated speck-like protein containing CARD (ASC), and inactive pro-Caspase-1. Finally, the assembly of inflammasome complexes leads to the cleavage and release of the active caspase-1 peptide, which further cleaves the signal peptide from pro-IL-1β, resulting in the generation of bio-active IL-1β and its release from the respective cell (18, 19).

Among the cell types reported to produce IL-1β, macrophages are of special importance because of their abundant tissue distribution and function as phagocytes (20). Besides, since macrophages have the ability to also act as APCs and trigger both innate and adaptive immune responses, it is of interest to study adjuvants for the activation of macrophages. It is known that several adjuvants are capable to activate the NLRP3 inflammasome, which is important for the adjuvant activity of for example aluminum, saponins, monophosphoryl lipid a (MPLA), and QS-21 (21, 22).

In the past few years, several publications have described how bacterial flagellins activate different immune cells (e.g., DCs and macrophages). However, the detailed mechanisms of how flagellin acts as an adjuvant to modulate immune responses are still not fully clear, especially when flagellin is fused with antigens, as in the investigated fusion protein. Furthermore, so far detailed mechanistic studies on how flagellin activates the inflammasome complex are limited, and the effects of flagellin-induced inflammasome activation on the production of other inflammatory cytokines need further investigation.

Our previous results suggested, that rFlaA:Betv1 can induce IL-1β secretion from macrophages (5), but the type of inflammasome activated, the underlying signaling mechanisms, and the contribution of inflammasome activation to the overall immune responses induced by rFlaA:Betv1 remained unknown. Therefore, the present study investigated the mechanisms contributing to rFlaA:Betv1-mediated IL-1β induction in both human and mouse macrophages, and the contribution of inflammasome activation and the produced IL-1β to the overall immune responses induced by rFlaA:Betv1.

## 2 Materials and methods

### 2.1 Generation of recombinant proteins

Recombinant flagellin A from *Listeria monocytogenes* (rFlaA, Acc. No: NC_003210) was generated according to (4), recombinant major birch pollen allergen Bet v 1 (Acc. No: X15877.1) according to (23). The fusion protein of rFlaA and rBet v 1 (rFlaA:Betv1) was generated according to (3) by cDNA fusion using the cDNAs of both rFlaA and rBet v 1 as templates. rFlaA^*D1^ and rFlaA^*D1^:Betv1 mutants were generated according to (15). For the generation of rFlaA^ΔDC0^ and rFlaA^ΔDC0^:Betv1 mutants, amino acids at position 251–287 of the *Listeria monocytogenes* flagellin A C terminal D0 domain, predicted to be involved in inflammasome activation of FliC (24), were deleted using a PCR mutagenesis strategy (Q5^®^ Site-Directed Mutagenesis Kit, NEB, Frankfurt, Germany). Protein expression and purification of the mutants was performed according to (15). The purity of all proteins was analyzed by SDS-PAGE (1 µg protein/lane) and Coomassie staining according to the method described by Laemmli (25) under non-reducing conditions (**Supplementary Figure 1A**). Moreover, the folding of secondary structure elements was determined by circular dichroism (CD)-spectroscopy (**Supplementary Figure 1B**). Purified proteins were adjusted to concentrations ranging from 210 to 320 µg/mL and dialyzed against 10 mM phosphate buffer. The spectra were recorded at ambient temperature (20°C) with a JASCO J-810 spectrophotometer (Jasco, Gross-Umstadt, Germany). Measurements were performed in a quartz glass cuvette (1 mm) with a step width of 1 nm and a band width of 1 nm. The spectral range was 185–255 nm at 50 nm/min. Ten scans were accumulated and spectra obtained with buffers were subtracted. The results were expressed as mean residue molar ellipticity [H]MRD as an indication of secondary structure element formation. The endotoxin content of each protein was assessed *via* chromogenic Limulus Amebocyte Lysate test according to the manufacturers’ instruction (Charles River, Sulzfeld, Germany): 0.16 pg/µg protein (rFlaA), 0.5 pg/µg protein (rBet v 1), 12.8 pg/µg protein (rFlaA:Betv1), <0.963 pg/µg protein (rFlaA^*D1^), 2.7 pg/µg protein (rFaA^*D1^:Betv1), 0.626 pg/µg protein (rFlaA^ΔDC0^), and 5.63 pg/µg protein (rFlaA^*D1^:Betv1) respectively (data not shown).

### 2.2 Cell culture

#### 2.2.1 Culture and stimulation of THP-1 cells

The wild type human monocyte cell line THP-1 was provided by Dr. Renate König (Paul-Ehrlich-Institut), NLRP3- (#thp-konlrp3z), NLRC4- (#thp-konlrc4z), and ASC-deficient (#thp-koascz) as well as NLRC4 overexpressing (#thp-nlrc4) THP-1 cells were purchased from *Invivo*Gen. THP-1 cells were maintained in RPMI1640 medium (Gibco, Karlsruhe, Germany), supplemented with 10% FCS (Sigma-Aldrich, Taufkirchen, Germany), 100 U/mL penicillin, 100 µg/mL streptomycin, 1 mM L-glutamine, 1 mM sodium pyruvate, 10 mM HEPES. Additional 100 μg/mL of Zeocin™ (*Invivo*Gen, Toulouse, France) was added in the medium used to cultivate the knockout cell lines. For THP-1 macrophage differentiation and stimulation, THP-1 monocytes were seeded at 3 x 10^5^ cells/mL in 24-well plates (Thermo Scientific, Dreieich, Germany) with culture medium plus 25 ng/mL PMA (Sigma-Aldrich) for 3 h. Subsequently, PMA-containing medium was discarded and replaced with fresh culture medium and incubated overnight at 37°C, 5% CO_2_. On the next day, cells were stimulated for 24 h with equimolar concentrations of either the indicated concentrations of the different proteins, LPS (#L5886, Sigma-Aldrich), ATP (#A9187, Sigma-Aldrich), or LPS plus ATP, which served as positive controls.

#### 2.2.2 Generation of human macrophages

Human macrophages were generated from buffy coats of anonymous blood donors according to (26).

#### 2.2.3 Isolation and stimulation of mouse peritoneal macrophages

C57BL/6J, TLR5^-/-^, and IL1R^-/-^ mice (C57BL/6J background, all from Jackson Laboratories, Bar Harbor, Maine, USA) were bred at the animal facility of the Paul-Ehrlich-Institut under specific pathogen-free conditions. After euthanizing animals with CO_2_, 10 mL of PBS supplemented with 3% FCS was injected into the peritoneal cavity, the fluid was collected again after gently shaking the mouse for 1 min. After centrifugation, cells were seeded at 5 x 10^5^ cells/500 μL in 24-well plates (Thermo Scientific) using RPMI 1640 supplemented with 10% FCS (Sigma-Aldrich), 100 U/mL penicillin, 100 µg/mL streptomycin, and 1 mM L-glutamine overnight. On the next morning, non-attached cells were washed again three times with PBS. The remaining attached peritoneal macrophages were stimulated with equimolar concentrations of the indicated proteins or LPS for either 24 h or 96 h.

#### 2.2.4 Culture and stimulation of HEK reporter cells

HEK-Blue™ hTLR5 cells (#hkb-htlr5) were purchased from *Invivo*Gen and maintained in DMEM medium (Gibco), supplemented with 10% FCS (Sigma-Aldrich), 100 U/mL penicillin, 100 µg/mL streptomycin, 1 mM L-glutamine, 30 µg/mL Blasticidin (*Invivo*gen), and 100 µg/ml Zeocin™ (*Invivo*Gen). For analysis, 2.5 x 10^4^ cells were seeded in HEK-Blue™ Detection Medium (#hb-det2, *Invivo*Gen) and treated with equimolar concentrations of either rFlaA, rFlaA^*D1^, rFlaA^ΔDC0^, rFlaA:Betv1, rFlaA^*D1^:Betv1, or rFlaA^ΔDC0^:Betv1 in 96-well plates (Thermo Scientific) for 16 h according to manufacturer’s recommendations. Hydrolysis of SEAP substrate was quantified using a SpectraMAX340PC (Molecular Devices, CA, USA) photometer at a wavelength of 635 nm.

### 2.3 Inhibitors and cell viability analysis

PMA-differentiated, THP-1 macrophages were pre-incubated with the indicated amounts of either the IKK-β inhibitors TPCA-1 (Abcam, Cambridge, UK) or BMS-345541 (Abcam), the unspecific inflammasome inhibitor VX-765 (*Invivo*Gen), which inhibits caspase-1 activity, the specific NLRP3-inflammasome inhibitor MCC950 (*Invivo*Gen), or the MAPK inhibitors SP600125 (SAP/JNK MAPK inhibitor, *Invivo*Gen), SB202190 (p38α/β MAPK inhibitor, *Invivo*Gen), or U0126 (ERK MAPK inhibitor, Cell Signaling Technologies, Leiden, The Netherlands) for 90 min and subsequently stimulated with rFlaA:Betv1 for 24 h. The target molecules of the used inhibitors are summarized in **Supplementary Figure 2**. For viability analysis, cells were treated as indicated, stained for dead cells using the fixable viability dye eFlour780 (#65-0865-14, eBioscience), and measured using a BD LSRFortessa™ flow cytometer (BD Biosciences). Data were analyzed using FlowJo V.7 (Treestar Inc., Ashland, OR, USA) and GraphPad PRISM (GraphPad Software, San Diego, California, USA).

### 2.4 ELISA

Levels of cytokines secreted from human cells were measured using either the IL-6 ELISA Set (#555220, BD Biosciences, Heidelberg, Germany), the IL-1β ELISA Set (#557953, BD Biosciences), the TNF-α Standard ABTS ELISA Development Kit (#900-K25, Peprotech, Hamburg, Germany), or the IL-12 Standard ABTS ELISA Development Kit (#900-K96, Peprotech) according to manufacturer’s recommendations. Cytokines in the supernatants of mouse cell cultures were analyzed using the following antibody combinations: IL-1β (capture antibody: anti-IL-1β monoclonal mouse antibody (#14-7061-85, eBioscience, Frankfurt, Germany, 1:500) plus detection antibody: anti-IL-1β monoclonal mouse biotin-conjugated antibody (#13-7112-81, eBioscience, 1:500)), IL-6 (capture antibody: anti-IL-6 monoclonal mouse antibody (#14-7061-85, eBioscience, Frankfurt, Germany, 1:500) plus detection antibody: anti-IL-6 monoclonal mouse biotin-conjugated antibody (#13-7062-85, eBioscience, 1:500), TNF-α (capture antibody: anti-TNF-α monoclonal mouse antibody (#14-7325-85, eBioscience, 1:500) plus detection antibody: anti-TNF-α monoclonal mouse biotin-conjugated antibody (#13-7326-85, eBioscience, 1:500), IL-12 p70 (capture antibody: anti-IL-12 monoclonal mouse antibody (#14-7122-85, eBioscience, 1:500) plus detection antibody: anti-IL-12 monoclonal mouse biotin-conjugated antibody (#MM121B, Invitrogen, 1:500). Following incubation with the respective detection antibodies, plates were incubated with 50 µL of diluted streptavidin horseradish peroxidase (#554066, eBioscience, 1:2000) for 30 minutes at room temperature. Detection was performed with 100 µL 3,3’,5,5’-tetramethylbenzidine (Carl Roth Chemikalien, Karlsruhe, Germany) and incubation for 3 to 5 min at room temperature. The reaction was stopped with 100 µL per well of 1 M sulfuric acid (Carl Roth Laborbedarf). Optical density was measured at 450 nm using a SpectraMAX340PC photometer (Molecular Devices).

### 2.5 Analysis of ROS generation

Generation of reactive oxygen species (ROS) in PMA-differentiated THP-1 macrophages was analyzed 24 h post stimulation with either ATP, LPS, LPS plus ATP, rFlaA, or rFlaA:Betv1 using the CellROX Green Reagent according to the manufacturers recommendations (#C10444, ThermoFisher Scientific). Fluorescence intensities were quantified as mean fluorescence intensities of live THP-1 cells using a BD FACSymphony A3 flow cytometer and analyzed using FlowJo V.7 (Treestar Inc., Ashland, OR, USA).

### 2.6 Western blot

For Western blot analysis, 1.6 x 10^6^ THP-1 cells were PMA-differentiated into macrophages in 6-well plates (Thermo Scientific). On the next day, cells were incubated with or without the indicated inhibitors for 90 min in RPMI supplemented with 1% FCS (Sigma-Aldrich) and subsequently stimulated with equimolar amounts of the indicated proteins for 24 h. The supernatants were harvested, and proteins were precipitated by adding trichloroacetic acid (Sigma-Aldrich) to bring the final concentration of trichloroacetic acid to 10% (w/v) and stored at -80°C for at least one day. Subsequently, samples were thawed on ice and centrifuged at 14000 rpm, 4°C for 15 min, and the pellet was washed once with 400 µL ice-cold acetone (Merck, Darmstadt, Germany). Protein pellets were air-dried for 3 min at room temperature and dissolved with 45 µL ddH_2_O followed by adding 15 µL of 4-fold sample dye for further analysis. For the generation of cell lysates, cells were washed once with ice-cold PBS and lysed with 150 µL lysis buffer (62.5 mM Tris-HCl (pH 6.8), 2% w/v SDS, 10% glycerol, 50 mM DTT, 0.01% w/v bromophenol blue) for 10 min on ice. Target proteins in lysates and precipitated supernatants were separated by SDS-PAGE and transferred to nitrocellulose membranes. After blocking with 5% non-fat milk, the membranes were incubated with the following primary antibodies overnight at 4°C: anti-NLRC4 antibody (#12421, Cell Signaling Technologies), anti-Caspase-1 antibody (#3866, Cell Signaling Technologies), anti-β-Tubulin antibody (#5346, Cell Signaling Technologies), anti-ASC antibody (#AG-25B-0006-C100, Adipogen, Fuellinsdorf, Switzerland), anti-NLRP3 antibody (#AG-20B-0014-C100, Adipogen), anti-IL-1β antibody (#AF-201-NA, R&D Systems, Wiesbaden-Nordenstadt, Germany). Detection was performed with the HRP-conjugated secondary antibodies using Immobilon Crescendo Western HRP substrate (#WBLUR0500, Merck, Darmstadt, Germany), and images were captured with iBright™ CL1500 system (Thermo Fischer Scientific). Band intensities in Western blots were quantified with ImageJ software (imagej. nih.gov) as relative light unit (RLU) normalized to the loading control.

### 2.7 Statistical analysis

Statistical analysis was performed with GraphPad Prism v9 for Windows using 2-way ANOVA tests with confidence intervals adjusted for multiple comparisons according to either Turkey or Dunnet. For statistically significant results the following convention was used: * - p-value < 0.05, ** - p-value < 0.01, *** - p-value < 0.001.

## 3 Results

### 3.1 rFlaA:Betv1 induces IL-1β and inflammatory cytokine secretion from both mouse and human macrophages

**Figure 1:**
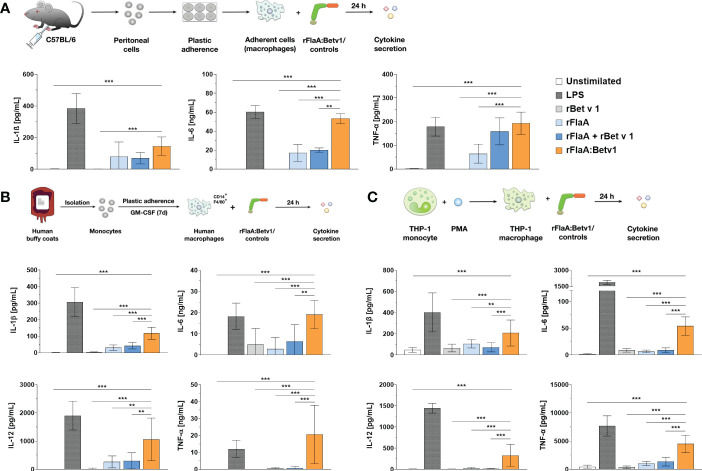
rFlaA: Betv1 induces IL-1β and pro-inflammatory cytokine secretion from both human and mouse macrophages. C57BL/6J peritoneal macrophages **(A)**, human buffy coat-differentiated macrophages **(B)**, or PMA-differentiated THP-1 macrophages **(C)** were stimulated with either LPS as a positive control or the indicated equimolar amounts of either rBet v 1, rFlaA, rFlaA + rBet v 1, or rFlaA:Betv1 for 24 h. Supernatants were collected and checked for the secretion of IL-1β, IL-6, IL-12, and TNF-α by ELISA. Data are the mean results ± SD from either three independent experiments **(A, C)** or samples collected from four donors **(B)**. Statistical significances are indicated as **: p-value < 0.01, ***: p-value < 0.001.

Our previous publication showed, that rFlaA:Betv1 induces a strong activation of *ex vivo* differentiated mouse BMDMs, leading to enhanced glucose metabolism and cytokine secretion, including IL-1β (5). To investigate whether rFlaA:Betv1 induced inflammasome activation, we first analyzed if rFlaA:Betv1 could induce IL-1β secretion from both mouse tissue-resident macrophages and human macrophages. For this, either mouse peritoneal macrophages isolated from C57BL/6J mice (**Figure 1A**), human buffy coat-differentiated macrophages (**Figure 1B**), or PMA-differentiated THP-1 macrophages (**Figure 1C**) were stimulated with either LPS as a positive control or equimolar amounts of rBet v 1, rFlaA, the mixture of rFlaA and rBet v 1 (rFlaA + rBet v 1), or the fusion protein rFlaA:Betv1 for 24 h, and analyzed for cytokine secretion (**Figure 1**). Here, rFlaA, rFlaA + rBet v 1, and rFlaA:Betv1 all resulted in induction of cytokine secretion from both mouse and human macrophages (**Figures 1A–C**). Furthermore, rFlaA:Betv1-stimulated peritoneal, buffy-coat-differentiated, and THP-1 macrophages showed significantly increased production of the inflammatory cytokines IL-1β, IL-6, and TNF-α compared to either unstimulated controls or cells stimulated with the single proteins alone (**Figure 1**). Higher IL-12 secretion from either human buffy coat- or PMA-differentiated THP-1 macrophages (**Figures 1B, C**) was also observed for the rFlaA:Betv1-stimulated groups. In contrast, no IL-12 secretion was detected from mouse peritoneal macrophages (data not shown). The results presented here suggest that the fusion protein rFlaA:Betv1 can activate different types of macrophages, including a prominent induction of IL-1β secretion, which is typically generated by inflammasome activation. Interestingly, the overall activation pattern observed for the different stimuli was similar between human primary buffy coat-differentiated macrophages and THP-1 cell line-derived macrophages (**Figures 1B, C**). Therefore, in the following experiments, we mainly focused on using PMA-differentiated THP-1 macrophages as model system.

### 3.2 Induction of inflammasome activation by rFlaA:Betv1 in THP-1 macrophages is independent of both the TLR5-activating QRMRQLAV-motif and the flagellin A C-terminal D0 domain

**Figure 2:**
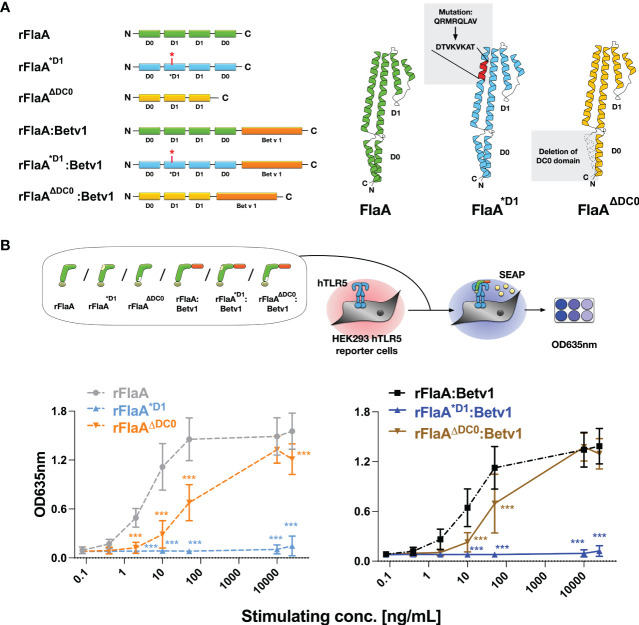
Generation and characterization of recombinant FlaA- and rFlaA:Betv1-mutants replacing either the TLR-activating sequence stretch in the D1 domain or truncating the C-terminal D0 domain. To investigate the mechanism of rFlaA:Betv1-mediated macrophage activation, four mutant proteins were generated **(A)**. For the generation of rFlaA^*D1^ and rFlaA^*D1^:Betv1 eight amino acids of the flagellin D1 structural domain (QRMRQLAV) reported to contribute to recognition of flagellin by TLR5 were replaced with the sequence DTVKVKAT **(A)**. For rFlaA^ΔDC0^ and rFlaA^ΔDC0^:Betv1 the flagellin DC0 domain was deleted **(A)**. The ability of wild-type and protein mutants to activate human TLR5 was tested using HEK-Blue™ hTLR5 reporter cells **(B)**. Data are the mean results of three independent experiments ± SD. Statistical comparisons were performed between the wild-type proteins (rFlaA or rFlaA:Betv1) and respective mutants treated with equimolar stimulation doses and statistical significance was shown as ***: p-value < 0.001.

Previous studies have shown, that flagellin type C (FliC) of *Salmonella typhimurium* can be recognized by both the surface receptor TLR5 and the cytosolic receptor NLRC4 (27). Here, eight amino acids in the N-terminal D1 domain of FliC (QRVRELAV) were essential for binding to TLR5 (28), while the C-terminal DC0 domain was shown to be important for the activation of the NLRC4 inflammasome (29). To address whether the observed THP-1 macrophage activation and IL-1β secretion induced by the rFlaA:Betv1 fusion protein is dependent on either the described TLR5-activating QRVRELAV-motif or the C-terminal DC0 domain, we generated mutant versions of rFlaA and the fusion protein, rFlaA^*D1^ and rFlaA^*D1^:Betv1, replacing the QRMRQLAV-motif with the sequence DTVKVKAT in the flagellin D1 domain. This sequence was previously shown to diminish the ability of flagellin molecules to bind to TLR5 (30) (**Figure 2A**). Moreover, rFlaA^ΔDC0^ and rFlaA^ΔDC0^:Betv1 mutants were produced, in which the FlaA DC0 domain (described to induce NLRC4 inflammasome activation (29)) was deleted (**Figure 2A**). All generated proteins were of high purity with the expected molecular weights (rBet v 1: 20 kDa, rFlaA: 32.7 kDa, rFlaA^*D1^: 32.2 kDa, rFlaA^ΔDC0^: 28.7 kDa, rFlaA:Betv1: 50.27 kDa, rFlaA^*D1^:Betv1: 49.77 kDa, and rFlaA^ΔDC0^:Betv1: 46.2 kDa, **Supplementary Figure 1A**) and the formation of secondary structure elements (**Supplementary Figure 1B**). Upon stimulation of hHEK293 reporter cells stably expressing human TLR5, both rFlaA^*D1^ and rFlaA^*D1^:Betv1 failed to activate TLR5 signaling in all tested concentrations (**Figure 2B**) (0.08-25,000 μg/mL, normalized to equimolar amounts of rFlaA) while the non-mutated proteins rFlaA and rFlaA:Betv1 readily induced TLR5-activation. Interestingly, truncation of the FlaA DC0 domain in both flagellin and the fusion protein partially reduced activation of human TLR5 by both rFlaA^ΔDC0^ and rFlaA^ΔDC0^:Betv1 mutants compared to wild type proteins. Besides this reduction, dose-dependent TLR5 activation was still achieved with both mutants (**Figure 2B**).

**Figure 3:**
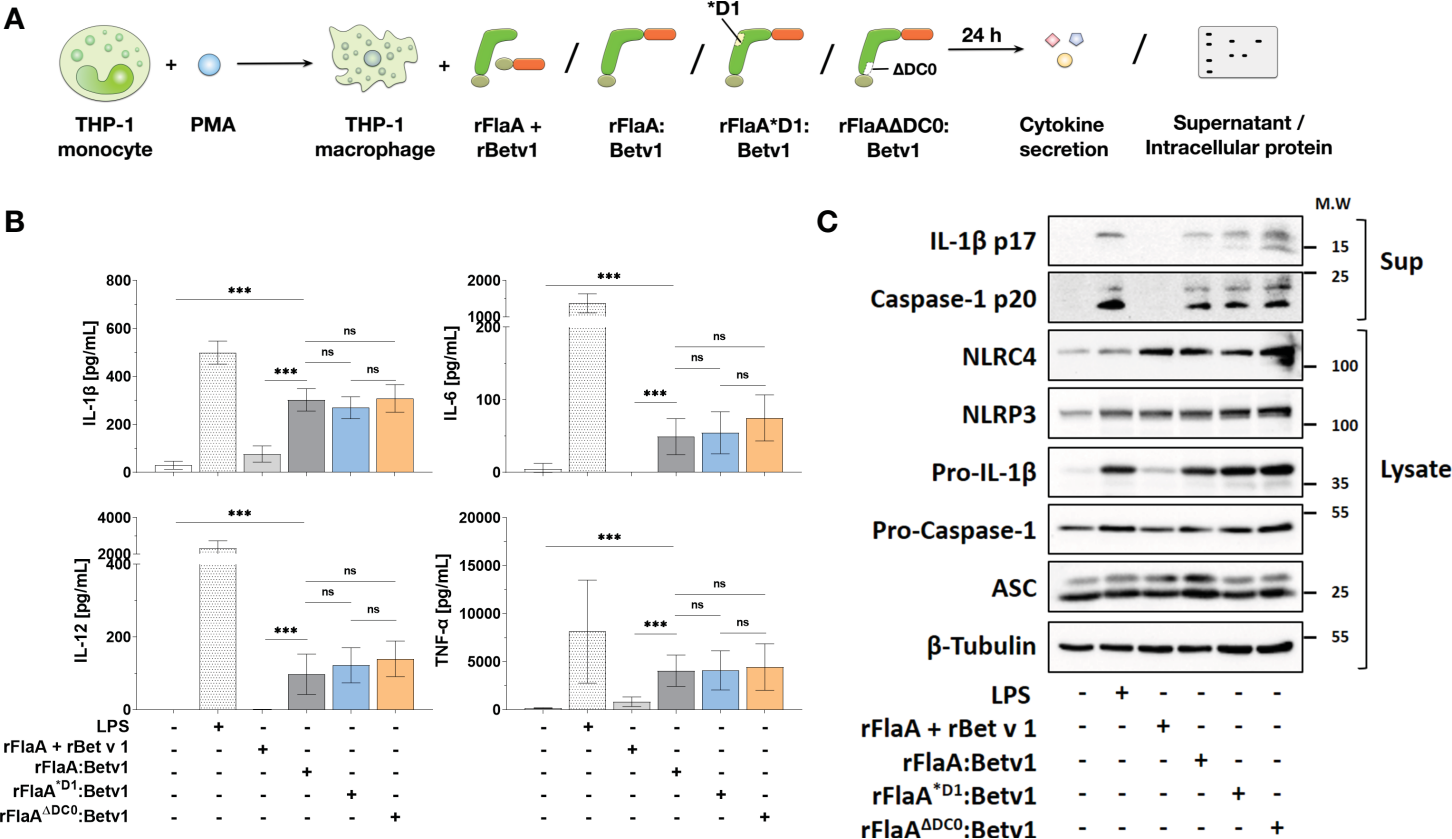
Cytokine secretion and induction of inflammasome activation by rFlaA:Betv1 in THP-1 macrophages is independent of both the TLR5-activating QRMRQLAV-motif and the flagellin C-terminal D0 domain. PMA-differentiated THP-1 macrophages were stimulated with either LPS as a positive control or equimolar amounts of rFlaA:Betv1, rFlaA^*D1^:Betv1, or rFlaA^ΔDC0^:Betv1 for 24 h **(A)**. Secreted cytokines were analyzed by ELISA **(B)** and proteins in lysates and precipitated supernatants were checked by Western Blot **(C)**. Data are either mean results ± SD **(B)** or the representative results **(C)** from three independent experiments with one lysate generated per experiment. Statistical significances are indicated as ns: p-value > 0.05, ***: p-value < 0.001.

We next applied rFlaA:Betv1 (and the mixture of both non-fused proteins), rFlaA^*D1^:Betv1, or rFlaA^ΔDC0^:Betv1 to PMA-differentiated THP-1 macrophages to see if the reduced TLR5-activation by the mutants resulted in differences in cytokine production (**Figure 3A**). Here, we observed no differences between the mutants and the unmodified fusion protein in either IL-1β-, IL-6-, TNF-α-, or IL-12-secretion from THP-1 macrophages (**Figure 3B**), indicating that the observed cellular activation by rFlaA:Betv1 is independent of both the TLR5-activating QRMRQLAV-motif and the FlaA DC0 domain.

Next, we performed Western Blot analyses to explore if the observed rFlaA:Betv1-induced IL-1β secretion by THP-1 macrophages is due to inflammasome activation (**Figure 3A** and **Supplementary Figure 3**). Here, both the classical NLRP3-stimulator LPS and rFlaA:Betv1, but not the mixture of rFlaA + rBet v 1, induced a time-dependent (between 6 to 24 h) activation of the inflammasome, which was confirmed by the detection of both cleaved forms of IL-1β (17 kDa) and caspase-1 (20 kDa) in the supernatant (**Supplementary Figure 3**). Besides, the results showed that LPS-, rFlaA:Betv1-, and two mutant fusion proteins induced higher pro-IL-1β protein expression in the cytosol compared to either unstimulated controls or the mixture of rFlaA + rBet v1, while also increasing the amount of the cleaved forms of IL-1β and caspase-1 in the supernatant (**Figure 3C**). Treatment with rFlaA + rBet v 1 also resulted in a slight induction of pro-IL-1β protein expression, but cleaved-IL-1β and caspase-1 were almost not detected in the respective supernatants (**Figure 3C**).

We further directly compared the IL-1β secretion induced by ATP alone as control, the NLRP3-inflammasome activators LPS or LPS + ATP, as well as the NLRC4 activator rFlaA, and rFlaA:Betv1 in THP-1 cells (**Supplementary Figure 4**). Here, both rFlaA and rFlaA:Betv1 induced lower but significant and still comparable levels of IL-1β secretion to the well-established NLRP3-activators LPS/ATP (**Supplementary Figure 4**). Using both an LPS titration (**Supplementary Figures 5A, B**) and LPS-controls that reflected the residual amounts of LPS contained within the stimulation concentrations of rFlaA:Betv1 used by us in this study (**Supplementary Figures 5A, C**), we could show that the minute LPS residues in our proteins induced substantially lower secretion of the investigated cytokines.

Detection of ROS in stimulated THP-1 cells showed that all tested stimuli (ATP, LPS, LPS + ATP, rFlaA, and the fusion protein rFlaA:Betv1) increased the production of ROS compared to unstimulated controls (**Supplementary Figure 6**). However, this increase was only found to be significant for rFlaA-stimulated THP-1 cells (**Supplementary Figure 6**).

In conclusion, the detection of cleaved-caspase-1 by Western Blot provided evidence that rFlaA:Betv1 can induce inflammasome activation in THP-1 macrophages.

### 3.3 Both NLRP3- and NLRC4-inflammasomes contribute to rFlaA:Betv1 induced IL-1β secretion and modulate the production of other inflammatory cytokines from THP-1 macrophages

**Figure 4:**
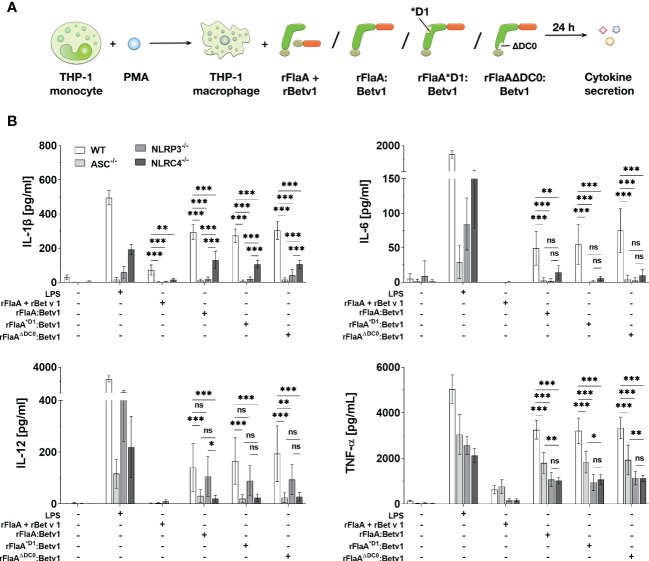
Both NLRP3- and NLRC4-inflammasome activation contributes to rFlaA:Betv1 induced IL-1β and pro-inflammatory cytokine secretion from THP-1 macrophages. PMA-differentiated wild-type (WT), NLRP3-, NLRC4-, or ASC-deficient THP-1 macrophages were stimulated with either LPS as a positive control, or equimolar amounts of rFlaA + rBet v 1, rFlaA:Betv1, rFlaA^*D1^:Betv1, or rFlaA^ΔDC0^:Betv1 for 24 h **(A)**. Supernatants were collected and checked for the secretion of IL-1β, IL-6, IL-12, and TNF-α by ELISA **(B)**. Data are the mean results of three independent experiments ± SD, and statistical significances are indicated as ns: p-value > 0.05, *: p-value < 0.05, **: p-value < 0.01, ***: p-value < 0.001.

To further address which type of inflammasome was activated by the flagellin:antigen fusion protein, wild type (WT), ASC-, NLRP3-, or NLRC4-deficient PMA-differentiated THP-1 macrophages were stimulated with either LPS as a positive control, equimolar amounts of rFlaA + rBet v 1, rFlaA:Betv1, rFlaA^*D1^:Betv1, or rFlaA^ΔDC0^:Betv1 for 24 h (**Figure 4A**). Here, the mixture of both proteins induced a low but detectable secretion of IL-1β and TNF-α from WT macrophages, whereas IL-6 and IL-12 were not detectable by ELISA (**Figure 4B**). Furthermore, rFlaA + rBet v 1-induced IL-1β production was shown to be ASC-, and interestingly both NLRP3- and NLRC4-inflammasome dependent (**Figure 4B**). In contrast, when stimulated with rFlaA + rBet v 1, ASC-deficient macrophages showed the same level of TNF-α secretion as WT controls (WT: 626.96 ± 182.14 pg/ml, ASC^-/-^: 766.38 ± 299.09 pg/mL), while only low levels of TNF-α were detected in either NLRP3- or NLRC4-knock out macrophages (NLRP3^-/-^: 167.44 ± 66.87 pg/mL, NLRC4^-/-^: 169.50 ± 53.65 pg/mL) (**Figure 4B**).

Compared to the mixture of both proteins, rFlaA:Betv1-stimulated WT THP-1 macrophages showed substantial higher secretion of all investigated cytokines (**Figure 4B**). Here, rFlaA:Betv1-induced IL-1β secretion was shown to be significantly dependent on inflammasome activation, as almost no IL-1β secretion was detected from ASC-deficient THP-1 macrophages (WT: 294.65 ± 42.45 pg/mL vs. ASC^-/-^: 10.38 ± 9.36 pg/mL) (**Figure 4B**). Interestingly, the rFlaA:Betv1-induced IL-1β secretion was completely abolished in NLRP3^-/-^ THP-1 macrophages (20.50 ± 10.53 pg/mL), while NLRC4 only partially contributed to rFlaA:Betv1-mediated IL-1β production (130.67 ± 51.43 pg/mL) (**Figure 4B**).

To further confirm that our fusion protein rFlaA:Betv1 induces both NLPR3- and NLRC4-inflammasome activation, we performed two additional sets of experiments (**Supplementary Figure 7**). In the first set of experiments, PMA-differentiated THP-1 cells were pre-treated with either (I) the unspecific inflammasome inhibitor VX-765, which inhibits caspase-1 activity or (II) the specific NLRP3-inflammasome inhibitor MCC950 (**Supplementary Figure 7A**). Interestingly, both inhibitors were able to suppress rFlaA:Betv1-induced IL-1β secretion (**Supplementary Figure 7B**), suggesting that the NLRP3 inflammasome is involved in the rFlaA:Betv1 induced macrophage activation.

Moreover, in a second set of experiments, stimulation of THP-1 cells stably overexpressing NLRC4 strongly enhanced rFlaA:Betv1-induced IL-1 β secretion compared to wild type cells (**Supplementary Figure 7C**), suggesting rFlaA:Betv1-mediated NLRC4 inflammasome activation to contribute to the observed IL-1β secretion.

When investigating cytokines other than IL-1β, interestingly, rFlaA:Betv1-induced IL-6 secretion was abrogated in all investigated knockout THP-1 macrophages, while LPS-induced IL-6 production was reduced to different extents in the different knockout THP-1 cells (**Figure 4B**). In contrast, both NLRC4- and NLRP3-inflammasomes were shown to partially contribute to rFlaA:Betv1-induced TNF-α secretion with deletion of ASC reducing TNF-α secretion by 45% and deletion of either NLRP3 or NLRC4 reducing TNF-α secretion by 67% and 69%, respectively.

Interestingly, only NLRC4 contributed to IL-12 secretion in rFlaA:Betv1-stimulated macrophages (**Figure 4B**). In addition, all results for the two proteins mutants rFlaA^*D1^:Betv1 and rFlaA^ΔDC0^:Betv1 were comparable to wild type rFlaA:Betv1, echoing our findings in **Figure 3**.

In summary, the analysis of rFlaA:Betv1-mediated cytokine secretion from ASC-, NLRP3-, or NLRC4-deficient THP-1 macrophages showed that our flagellin:antigen fusion protein could induce dual NLRP3- and NLRC4-inflammasome activation. Interestingly, both inflammasomes also contributed (either fully or only in part) to the secretion of other inflammatory cytokines (IL-6 and TNF-α).

### 3.4 NFκB- and SAP/JNK MAP kinase-signaling regulate rFlaA:Betv1-induced inflammasome activation by modulating pro-Caspase-1- and pro-IL-1β-expression in THP-1 macrophages

To further analyze the intracellular signaling pathways that contribute to the observed activation of THP-1 macrophages by rFlaA:Betv1, cells were stimulated with either LPS, rBet v 1, rFlaA, rFlaA + rBet v 1, or rFlaA:Betv1 for 30 minutes and examined for activation of MAPK- and NFκB-pathways by Western Blot (**Supplementary Figure 8A**). Both LPS and rFlaA:Betv1 induced a significant p38- and SAP/JNK-MAPK phosphorylation compared to either unstimulated controls or cells stimulated with both proteins alone or as a mixture (**Supplementary Figures 8B, C**). Moreover, both IKK and NF*κ*B subunit p65 were found to be phosphorylated, paralleling the downregulation of I*κ*Bα levels (**Supplementary Figures 8B, C**). Stimulation with either rFlaA or rFlaA + rBet v 1 also resulted in a slight induction of MAPK- and NFκB-signaling pathways compared to the unstimulated group (**Supplementary Figures 8B, C**). In contrast, rBet v 1 induced no changes in the investigated protein levels or their phosphorylation pattern (**Supplementary Figures 8B, C**). We also observed basal, high-level phosphorylation of ERK-MAPK in THP-1 cells, which was not changed upon treatment with the different stimuli (**Supplementary Figures 8B, C**). Therefore, in the following experiments, we excluded the analyses of ERK-MAPK when investigating rFlaA:Betv1 induced THP-1 macrophage activation.

**Figure 5:**
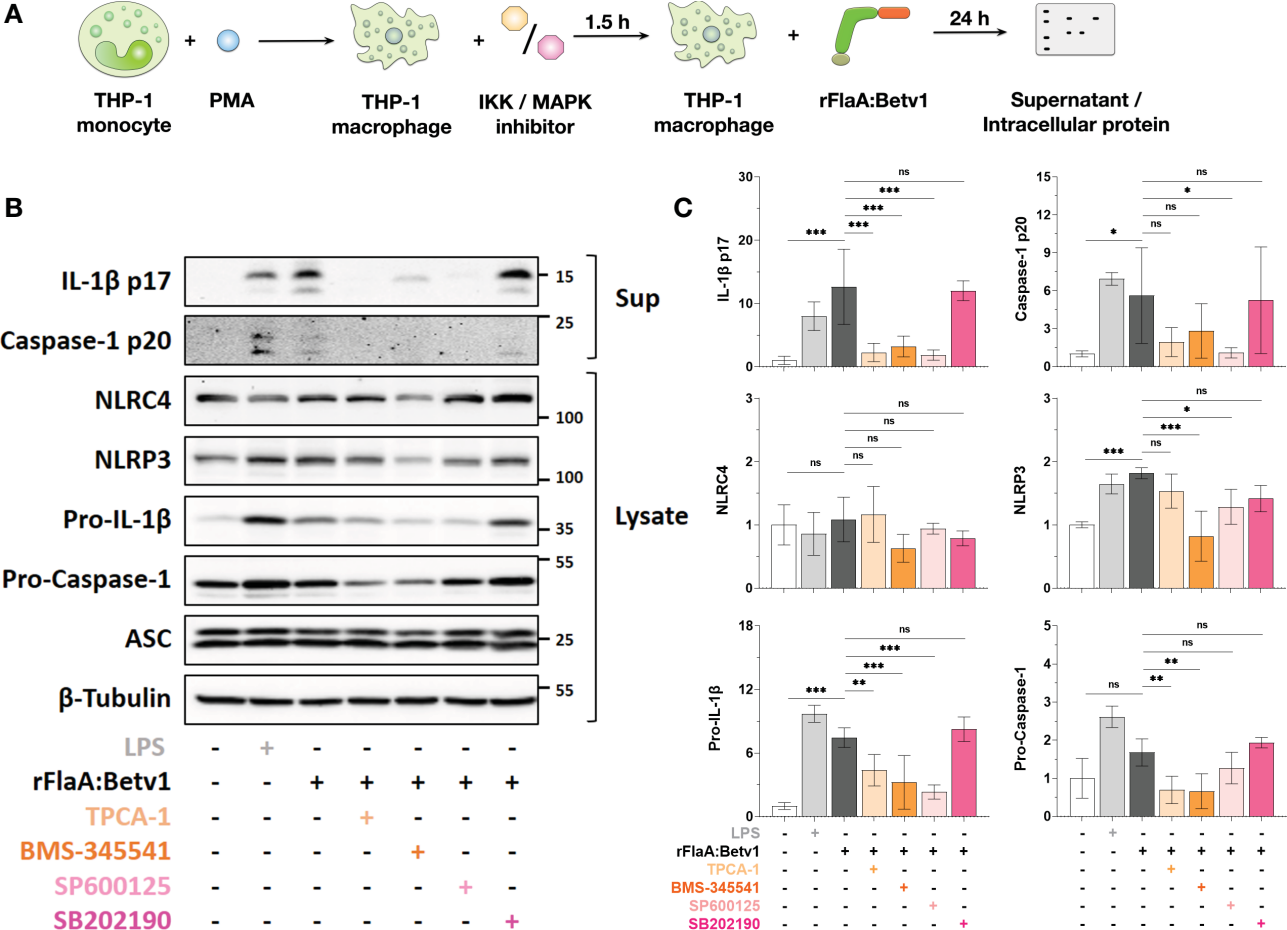
NFκB- and SAP/JNK MAP kinase-signaling pathways contribute to inflammasome activation by regulating pro-Caspase-1, pro-IL-1β-, and NLRP3-expression in rFlaA:Betv1-stimulated THP-1 macrophages. PMA-differentiated THP-1 macrophages were pre-treated with the indicated inhibitors (500 nM TPCA-1, 5 µM BMS-345541, 25 µM SP600125, or 10 µM SB202190) for 90 minutes, followed by stimulation with 27.4 µg/mL rFlaA:Betv1 for additional 24 h **(A)**. Proteins in supernatant (Sup) and cell lysate (Lysate) were examined by Western Blot **(B)**. The intensity of Western Blot bands from three independent experiments was analyzed, first normalized to the loading control β-Tubulin and then again normalized to the unstimulated group (shown as value of “1”) **(C)**. Data are either representative **(B)** or mean results of three independent experiments ± SD **(C)**. Statistical significances are indicated as ns: p-value > 0.05, *: p-value < 0.05, **: p-value < 0.01, ***: p-value < 0.001.

To further dissect the contribution of MAPK- and NF*κ*B-signaling to rFlaA:Betv1-induced inflammasome activation, THP-1 macrophages were pre-treated with inhibitors of either IKK- (TPCA-1 or BMS-345541), SAP/JNK MAPK- (SP600125), or p38 MAPK-activation (SB202190) (modes of action of the different inhibitors shown in **Supplementary Figure 2**) for 90 min, followed by stimulation with rFlaA:Betv1 for additional 24 h. Subsequently, we analyzed the expression of proteins involved in inflammasome activation from cell lysates and the cleaved forms of IL-1β and caspase-1 in the supernatants (**Figure 5A**). Cytotoxic effects of the applied inhibitor concentrations on the THP-1 macrophages were excluded by live-dead staining (**Supplementary Figure 9**).

Here, both IKK inhibitors TPCA-1 and BMS-345541 significantly inhibited rFlaA:Betv1-induced IL-1β p17- (by 82% for TPCA-1 and 75% for BMS-345541), and caspase-1 p20-levels (by 66% for TPCA-1 and 50% for BMS-345541) in the supernatant (**Figures 5B, C**). Similarly, the SAP/JNK MAPK-inhibitor SP600125 suppressed IL-1β p17 secretion by 85% and caspase-1 p20 by 81%, while inhibition of p38 MAPK by SB202190 did not affect the secretion of both proteins from rFlaA:Betv1-stimulated THP-1 macrophages (**Figures 5B, C**).

Upon rFlaA:Betv1 stimulation in THP-1 macrophages, the two NLR family members, NLRC4 and NLRP3, were differentially regulated by MAPK- and NF*κ*B-signaling. Here, suppression of these two pathways did not affect cytosolic NLRC4 expression. However, both LPS- and rFlaA:Betv1-stimulation induced upregulation of NLRP3, while pre-incubation with either BMS-345541 or SP600125 significantly suppressed NLRP3-induction (**Figures 5B, C**).

Furthermore, a significant increase in levels of pro-IL-1β in cell lysates was observed in either LPS- or rFlaA:Betv1 stimulated groups, and pre-treatment with both IKK-inhibitors TPCA-1 or BMS-345541, as well as the SAP/JNK MAPK-inhibitor SP600125 significantly suppressed pro-IL-1β expression by 41%, 57%, and 69% respectively (**Figures 5B, C**). In contrast, only the NFκB-pathway contributed to the slight induction of pro-Caspase-1 expression from rFlaA:Betv1-stimulated THP-1 macrophages (**Figures 5B, C**). In line with the result, that IL-1β p17 and caspase-1 p20 secretion were not affected by pre-incubation with the p38 MAPK inhibitor SB202190, expression levels of all proteins tested in the cell cytosol remained unchanged under these conditions (**Figures 5B, C**).

Taken together, MAPK- and NFκB-signaling pathways were shown to contribute to rFlaA:Betv1-induced inflammasome activation in THP-1 macrophages by regulating the expression of proteins comprising the inflammasome complex in different ways.

### 3.5 The NFκB-signaling pathway regulates rFlaA:Betv1-induced cytokine secretion from THP-1 macrophages

**Figure 6:**
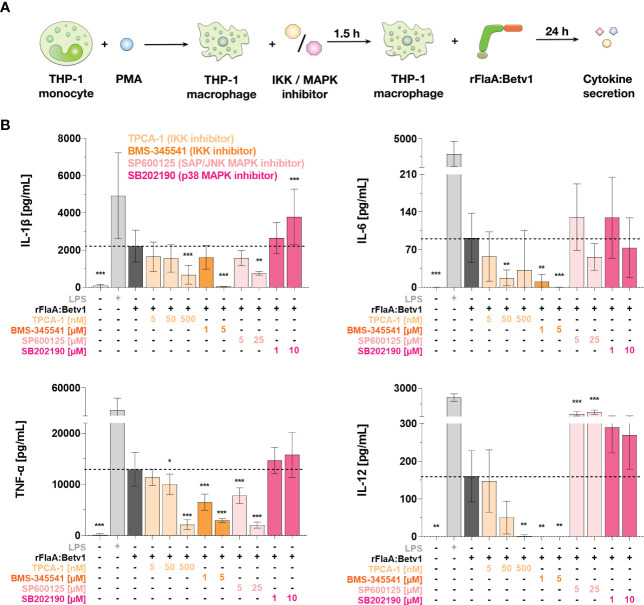
NFκB- and SAP/JNK MAP kinase-signaling pathways contribute to rFlaA:Betv1-induced cytokine secretion from THP-1 macrophages. PMA-differentiated THP-1 macrophages were pre-treated with the indicated inhibitor concentrations for 90 min and subsequently stimulated with 27.4 µg/mL rFlaA:Betv1 for additional 24 h **(A)**. Supernatants were collected and examined for the secretion of IL-1β, IL-6, IL-12, and TNF-α by ELISA **(B)**. Data are the mean results of three independent experiments ± SD. Statistical comparisons were performed between the indicated samples and rFlaA:Betv1-stimulated samples, with statistical significance shown as *: p-value < 0.05, **: p-value < 0.01, ***: p-value < 0.001.

We next investigated the contribution of both MAPK- and NFκB-signaling pathways to rFlaA:Betv1-induced cytokine secretion from THP-1 macrophages. For this, we used the same set of inhibitors described above (**Figure 6A** and **Supplementary Figure 9**). In this experimental setup, the inhibition of IKK by either TPCA-1 or BMS-345541 dose-dependently and highly significantly suppressed the secretion of all investigated, rFlaA:Betv1-induced cytokines (IL-1β, IL-6, TNF-α, and IL-12, **Figure 6B**). On the contrary, pre-incubation with the SAP/JNK MAPK-inhibitor SP600125 only inhibited rFlaA:Betv1-induced IL-1β- and TNF-α-secretion by 66% and 84% in the highest concentration respectively, while IL-12 secretion was significantly increased (4.5-fold increase compared to rFlaA:Betv1-stimulated cells) and IL-6 remained unaffected (**Figure 6B**). Moreover, the p38 MAPK inhibitor SB202190 had again no effect on rFlaA:Betv1-induced cytokine secretion from THP-1 macrophages (**Figure 6B**).

To sum up, NFκB activation significantly contributes to rFlaA:Betv1-mediated secretion of inflammatory cytokines from THP-1 macrophages. With regard to MAPK-signaling, only SAP/JNK MAPK-signaling contributed to IL-1β- and TNF-α-secretion upon rFlaA:Betv1 stimulation, while no contribution of p38 MAPK-activation was observed.

### 3.6 The IL-1β-IL1R1 autocrine pathway contributes to rFlaA:Betv1-induced IL-1β-, IL-6-, and TNF-α-secretion from mouse peritoneal macrophages

**Figure 7:**
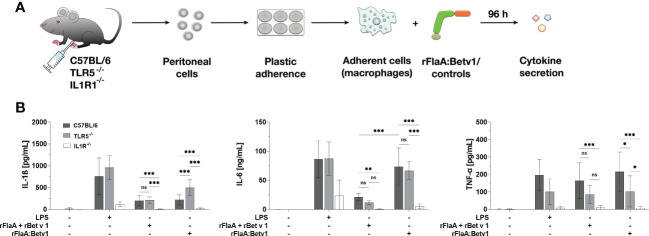
rFlaA:Betv1 induced IL-1β-, IL-6-, and TNF-α-secretion is dependent on the IL-1β-IL1R1 autocrine pathway in mouse peritoneal macrophages. Peritoneal macrophages isolated from either C57BL/6J-, TLR5^-/–^, or IL1R^-/–^mice were stimulated with either LPS as a positive control or equimolar amounts of either rFlaA + rBet v 1 or rFlaA:Betv1 for 96 h **(A)**. Supernatants were collected and checked for the secretion of IL-1β, IL-6, and TNF-α by ELISA **(B)**. Data are the mean results of three independent experiments ± SD, and statistical significances are indicated as ns: p-value > 0.05, *: p-value < 0.05, **: p-value < 0.01, ***: p-value < 0.001.

Previous literature has shown, that IL-1β can induce autocrine immune cell activation *via* IL1R1 thereby promoting further IL-1β secretion in a positive feedback loop in nucleated cells (31). Besides, IL-1β binding to IL1R also induces MAPK- and NFκB-activation, which can modulate immune cell activation (32). To verify whether the IL-1β-IL1R1 autocrine pathway also contributes to the secretion of inflammatory cytokines from rFlaA:Betv1-stimulated macrophages, peritoneal macrophages from either C57BL/6J wild type, TLR5-, or IL1R1-deficient mice were isolated and stimulated with either the fusion proteins or the respective controls, and then analyzed for cytokine secretion (**Figure 7A**).

Here, secretion of IL-1β and IL-6 induced by either the positive control LPS, rFlaA + rBet v 1, or rFlaA:Betv1 was shown to be TLR5-independent, whereas secretion of TNF-α in the rFlaA:Betv1-stimulated group was shown to be partially TLR5-dependent (**Figure 7B**). Besides, rFlaA:Betv1-induced IL-1β production was increased in TLR5-deficient macrophages compared to wild type controls (**Figure 7B**). Interestingly, no secretion of the investigated cytokines was detected from IL1R1-deficient peritoneal macrophages for all tested stimuli (**Figure 7B**). These results suggest the IL-1β-IL1R1 autocrine pathway to be essential for rFlaA:Betv1-induced secretion of inflammatory cytokines from (mouse peritoneal) macrophages.

## 4 Discussion

Our previous publications showed rFlaA:Betv1 to activate different cell types involved in allergic responses, resulting in the suppression of allergen-induced Th2 inflammation (3, 5–7, 14, 15). In these studies, the rFlaA:Betv-induced IL-1β secretion was shown to be differently regulated by different types of APCs, including mDCs and BMDMs (3, 5, 14). Since the canonical production of IL-1β results from the formation of inflammasome complexes (19), and the activation of inflammasomes is important for the immunogenicity of many different adjuvants (21, 22), in this study we investigated the mechanisms underlying macrophage activation by the rFlaA:Betv1 fusion protein, with a particular focus on the signaling pathways contributing to inflammasome activation and their effect on cytokine secretion. Although it is well known that both bacterial flagellin and components of the type III secretion system can activate the NLRC4 inflammasome (19), the detailed signaling pathways by which flagellin:antigen fusion proteins can activate the inflammasome are still not fully understood.

Therefore, in our study, we used a flagellin-allergen fusion protein (rFlaA:Betv1) as a model for flagellin-adjuvanted future vaccines and therapeutics and found for the first time that flagellin:allergen fusion proteins can induce dual NLRP3- and NLRC4-inflammasome activation. Moreover, rFlaA:Betv1-activated NFκB- and MAPK-pathways were found to regulate the expression of different proteins involved in inflammasome complex formation. Most importantly, we demonstrated that both rFlaA:Betv1-induced inflammasome activation and IL-1β signaling (in a positive feedback loop *via* the IL-1R) are also required for the secretion of other pro-inflammatory cytokines (e.g. IL-6, IL-12, and TNF-α) by macrophages. We hope these new findings will provide valuable insights for future vaccine and adjuvant development.

### 4.1 rFlaA:Betv1 induces activation of both mouse tissue-resident macrophages and human macrophages

Our previous study analyzed how rFlaA:Betv1 activated *ex vivo*-differentiated mouse BMDMs, showing that rFlaA:Betv1 triggered a strong MyD88-dependent, but mostly TLR5-independent cytokine production and activation of glycolytic metabolism in BMDMs (5). Besides, rFlaA:Betv1-stimulation induced a strong activation of HIF-1α-, MAPK-, and NF*κ*B-signaling in BMDMs (5). While the observed anti-inflammatory IL-10- and pro-inflammatory IL-6- and TNF-α-secretion were either partially dependent on mTOR- or fully dependent on SAP/JNK MAPK-activation (5), the induced secretion of IL-1β from BMDMs by rFlaA:Betv1 was not dependent on any of the pathways we previously analyzed (5). Moreover, the effect of rFlaA:Betv1 on either mouse tissue-resident macrophages or human macrophages remained unknown. Therefore, in this study, we firstly demonstrated that rFlaA:Betv1 induced strong pro-inflammatory cytokine secretion, including IL-1β, from *ex vivo*-isolated mouse peritoneal macrophages, human buffy-coat-differentiated macrophages, and PMA-differentiated THP-1 macrophages when treated with either both proteins alone or as a mixture. These results are comparable to our previously published findings from BMDMs (5), and are also the first evidence showing that flagellin:antigen fusion protein can induce human macrophage activation.

### 4.2 rFlaA:Betv1 induces dual NLRP3- and NLRC4-inflammasome activation

Flagellin can be recognized by both TLR5 on the cell surface and NLRC4 in the cytosol (1, 33). Several studies have revealed a sequence stretch of eight highly conserved amino acids in flagellins N-terminal D1 domain (QRVRELAV) to be important for binding to TLR5 (28, 30, 34). The conserved TLR signaling pathway is known for activating NFκB- and MAPK-pathways, thereby promoting cytokine secretion and pro-inflammatory gene expression in immune cells (35). In addition, the canonical production of bioactive IL-1β is dependent on additional inflammasome complex formation (18, 19). In this context, flagellin is known to trigger NLRC4 inflammasome activation, since either deletion or mutation of flagellin FliC from *Salmonella typhimurium* or *Legionella pneumophila* abolished NLRC4 inflammasome activation in mouse macrophages upon bacterial infection (36, 37). Further studies revealed, that 35 amino acids in the C-terminal DC0 domain of *Salmonella typhimurium* flagellin are critical for the induction of caspase-dependent cell death of mouse macrophages (29, 38).

To verify the role of either the TLR5-binding motif in the D1 domain or the NLRC4-activating C-terminal DC0 domain of *Listeria monocytogenes* flagellin A in the rFlaA:Betv1-induced activation of human macrophages, and according to our initial aim to dissect the contribution of either NLRC4- or TLR5-activation to rFlaA:Betv1-mediated macrophage activation, we generated both flagellin and flagellin:antigen fusion proteins mutants. Our results showed, that neither rFlaA^*D1^ or rFlaA^*D1^:Betv1 were able to activate human TLR5-signaling, and interestingly rFlaA^ΔDC0^ and rFlaA ^ΔDC0^:Betv1 also displayed a partially reduced TLR5 activation. These results are in line with the publication by Forstnerič et al., in which the authors demonstrated that the DC0 domain of *Salmonella typhimurium* flagellin is required for TLR5-activation (39). However, when applying the mutants on THP-1 macrophages, our results showed that neither the TLR5-binding region nor the DC0 domain of FlaA were necessary for rFlaA:Betv1-induced cytokine secretion and inflammasome activation. This TLR5-independence is line with our previous findings showing the rFlaA:Betv1-mediated activation of mouse BMDMs, mDCs, epithelial cells, and B cells to be largely TLR5-independent (3, 5, 6, 15).

Since we observed rFlaA:Betv1-induced IL-1β secretion from THP-1 macrophages to be independent of the flagellin A DC0 domain, we hypothesized that other inflammasomes than the NLRC4 inflammasome might be activated by our fusion protein. Interestingly, by using THP-1, ASC-, NLRP3-, and NLRC4-knockout cells, we could demonstrate (**I**) the induced IL-1β production to be based on inflammasome activation, as IL-1β was not detectable in rFlaA:Betv1-stimulated ASC^-/-^ THP-1 cells, and (**II**) the induced IL-1β secretion to be strongly dependent on NLRP3 while showing only a partial dependence on the NLRC4 inflammasome. Similar to our results, several studies have shown that both NLRC4 and NLRP3 are required to induce BMDM-derived IL-1β production after *Salmonella typhimurium* infection (40, 41). In addition, activation of NLRC4 can recruit NLRP3 to co-localize with ASC and caspase-1 in the same inflammasome complex, resulting in the interaction between NLRP3 and the NACHT, also called NAIP (neuronal apoptosis inhibitor protein), domain of NLRC4 (40, 41). Moreover, activation of the NLRP3 inflammasome triggered by either bacterial infection, flagellin, or flagellin:antigen fusion proteins might be caused by mitochondrial instability, resulting in mitochondrial ROS production (42), or a shift in overall cell metabolism towards glycolysis, resulting in a disruption of the mitochondrial Krebs cycle, which we previously observed in the rFlaA:Betv1-stimulated BMDMs (5). While we observed both rFlaA and rFlaA:Betv1 to increase ROS production from THP-1 cells, this ROS production was only found to be significant for rFlaA-stimulated cells.

### 4.3 rFlaA:Betv1 induces NFκB- and SAP/JNK MAPK-activation which contribute to both inflammasome activation and cytokine secretion

Although the activation of NFκB-signaling by the TLR4 ligand LPS, inducing pro-IL-1β- and NLRP3-expression in order to initiate NLRP3 inflammasome activation, has been intensively studied (43), the detailed mechanisms of how either flagellin or flagellin:antigen fusion proteins contribute to inflammasome activation are not fully clear. Here we demonstrated, that both rFlaA and rFlaA:Betv1 could induce activation of NFκB- and MAPK-signaling in THP-1 macrophages. And by using specific inhibitors, we showed that NFκB-activation contributed to rFlaA:Betv1-induced pro-IL-1β-, NLRP3- and pro-caspase-1-expression, while SAP/JNK MAPK-activation mediated pro-IL-1β- and NLRP3-expression. Furthermore, blockade of the NFκB pathway was shown to abrogate rFlaA:Betv1-induced secretion of pro-inflammatory IL-6, IL-12, and TNF-α from THP-1 macrophages, whereas blockade of SAP/JNK MAPK affected only IL-1β and TNF-α secretion. Moreover, inhibition of p38 MAPK activation did not affect expression of the investigated proteins, which is in line with our previous study in BMDM where blocking p38 MAPK activation did not affect rFlaA:Betv1-induced IL-1β secretion (5). Interestingly, our previous findings showed no contribution of SAP/JNK MAPK to rFlaA:Betv1-induced IL-1β secretion in mouse BMDMs (5), but in the present study we found that SAP/JNK MAPK signaling plays an important role in mediating inflammasome activation in human THP-1 macrophages. These differences may be due to the fact that different species and types of macrophages differentially regulate the activation of MAPK pathways (44).

### 4.4 The IL-1β-IL1R1 feedback loop contributes to rFlaA:Betv1-induced cytokine production by macrophages

The main role of the inflammasome is to induce the secretion of the pro-inflammatory cytokines IL-1β and IL-18, as well as to induce pyrolysis of immune cells, which further triggers and potentiates the induced immune responses (19). The produced IL-1β can bind to its receptor IL1R1 and recruit the adaptor protein MyD88, which leads to the activation of several signaling pathways including NFκB- and MAPK-mediated signaling events, that regulate inflammatory gene expression and production of cytokines like IL-6, TNF-α, and IL-1α (45). In the present study, by using peritoneal macrophages isolated from IL1R1 knock-out mice, we demonstrated that the IL-1β-IL1R1 feedback loop is important for rFlaA:Betv1-induced cytokine secretion from mouse peritoneal macrophages. Interestingly the overall induced IL-1β and IL-6 secretion were shown to be TLR5-independent, which is comparable to our findings when treating THP-1 macrophages with the rFlaA^*D1^:Betv1 mutant. Moreover, our previous study showed that rFlaA:Betv1-induced BMDM activation was MyD88-dependent but TLR5-independent, and rFlaA:Betv1 also still induced NFκB- and MAPK-activation in TLR5-deficient macrophages (5). Since TLR- and IL-1R1-activation can both recruit MyD88, our results on the contribution of the IL-1β-IL1R1 loop to macrophage activation may explain the observed TLR5-independence upon rFlaA:Betv1 stimulation.

Interestingly, rFlaA:Betv1-induced IL-β secretion was significantly enhanced in TLR5-deficient peritoneal macrophages. These results may be explained by the findings of Carvalho et al.: They could show that TLR5-deficiency resulted in decreased production of the secretory interleukin-1 receptor antagonist (sIL1Ra) *in vitro* in intestinal epithelia and macrophages, which counteracted the strongly pro-inflammatory actions of IL-1β (46). Therefore, loss of TLR5 may promote the observed increase macrophage-derived IL-1β secretion.

### 4.5 NLRP3 and NLRC4 differentially regulate rFlaA:Betv1-triggered inflammatory cytokine secretion

Although it is well known that activation of all types of inflammasomes leads to the production of IL-1β and IL-18 (19), the effect of different NOD-like receptors on the secretion of other inflammatory cytokines remains unclear. Since our rFlaA:Betv1 fusion protein induced dual activation of NLRP3- and NLRC4-inflammasomes in THP-1 macrophages, we also investigated whether NLRP3 and NLRC4 differentially regulated the secretion of other pro-inflammatory cytokines. First, rFlaA:Betv1-treated ASC knockout THP-1 cells showed reduced secretion of IL-6, TNF-α, and IL-12, suggesting that inflammasome activation regulated cytokine secretion. Concordantly, with the results discussed above, this inflammasome-dependency may be caused by either the positive feedback loop induced by binding of IL-1β to its receptor or the activation of MyD88-dependent signaling pathways, as Taxman and colleagues found that in ASC^-/-^ macrophages, reduced cytokine expression was associated with suppressed NFκB activity upon *Porphyromonas gingivalis* infection (47). Interestingly, we found that rFlaA:Betv1-induced secretion of IL-6 and TNF-α was dependent on both NLRP3 and NLRC4, whereas secretion of IL-12 was dependent only on NLRC4 in THP-1 macrophages. Here, the detailed mechanisms underlying this dual inflammasome activation remain to be revealed.

In summary, we demonstrated that flagellin, used as an adjuvant fused to the major birch pollen allergen Bet v 1, can induce dual activation of NLRP3- and NLRC4-inflammasomes in macrophages, leading to IL-1β secretion. In addition, NFκB-, SAP/JNK MAPK-signaling, and IL-1β-IL1R1 feedback loop were not only shown to be important factors in the regulation of rFlaA:Betv1-mediated macrophage activation and cytokine secretion but to also be tightly connected to inflammasome activation. These findings may help us to better understand the adjuvant properties of flagellin and develop future novel therapeutics or vaccines using flagellin as an adjuvant.

## Data availability statement

The raw data supporting the conclusions of this article will be made available by the authors, without undue reservation.

## Ethics statement

Ethical approval was not provided for this study on human participants because Cells were generated from commercially available Buffy coats. No patient data were collected. Written informed consent for participation was not required for this study in accordance with the national legislation and the institutional requirements.

## Author contributions

Y-JL: data curation, methodology, formal analysis, investigation, visualization, and writing – original draft & review & editing. SW and AF: methodology, formal analysis, and investigation. AJ and A-CJ: methodology, formal analysis, investigation, and writing – review & editing. AG: methodology and writing– review & editing. SSche: conceptualization, funding acquisition, supervision, and writing – review & editing. SSchü: funding acquisition, conceptualization, data curation, project administration, supervision, visualization, and writing – original draft & review & editing. All authors contributed to the article and approved the submitted version.
