# Supplementary material for: A flagellin-conjugate protein induces dual NLRC4- and NLRP3-inflammasome activation which modulates inflammatory cytokine secretion from macrophages
Source: Front Immunol. 2023 Mar 21;14:1136669. doi: 10.3389/fimmu.2023.1136669 (PMC10070734; doi:10.3389/fimmu.2023.1136669)
Supplement: Supplementary file 1 [file DataSheet_1.docx]

A flagellin-conjugate protein induces dual NLRC4- and NLRP3-inflammasome activation which modulates inflammatory cytokine secretion from macrophages

Yen-Ju Lin^1^, Annette Jamin^1^, Sonja Wolfheimer^1^, Anna Fiedler^1^, Ann-Christine Junker^1^, Alexandra Goretzki^1^, Stephan Scheurer^1^, Stefan Schülke^1*^

^1^Molecular Allergology, Paul-Ehrlich-Institut, Langen, Germany

*** Correspondence:**

Stefan Schülke, PhD

Phone: +49 6103 775209

Fax: +49 6103 771258

E-Mail: stefan.schuelke@pei.de

Supplementary Material

# Supplementary Figures

**
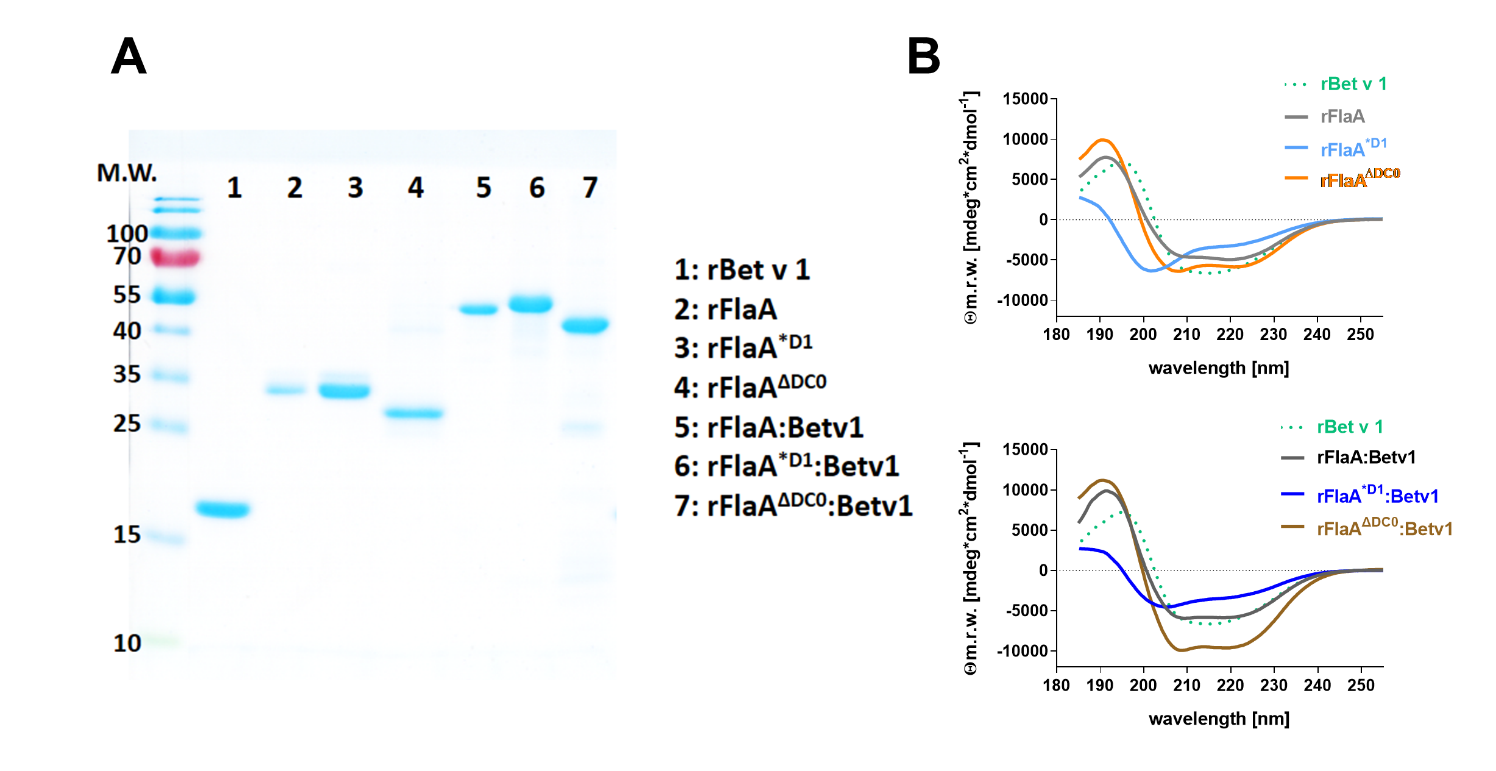
**

**Supplementary Figure 1. Characterization of purified recombinant rFlaA^*D1^, rFlaA^ΔDC0^, rFlaA^*D1^:Betv1, and rFlaA^ΔDC0^:Betv1 proteins.** Purity and molecular weight of the recombinant proteins were analyzed by SDS-PAGE with Coomassie staining **(A)**. The folding of secondary structure elements was examined by circular dichroism spectra **(B)**.

**
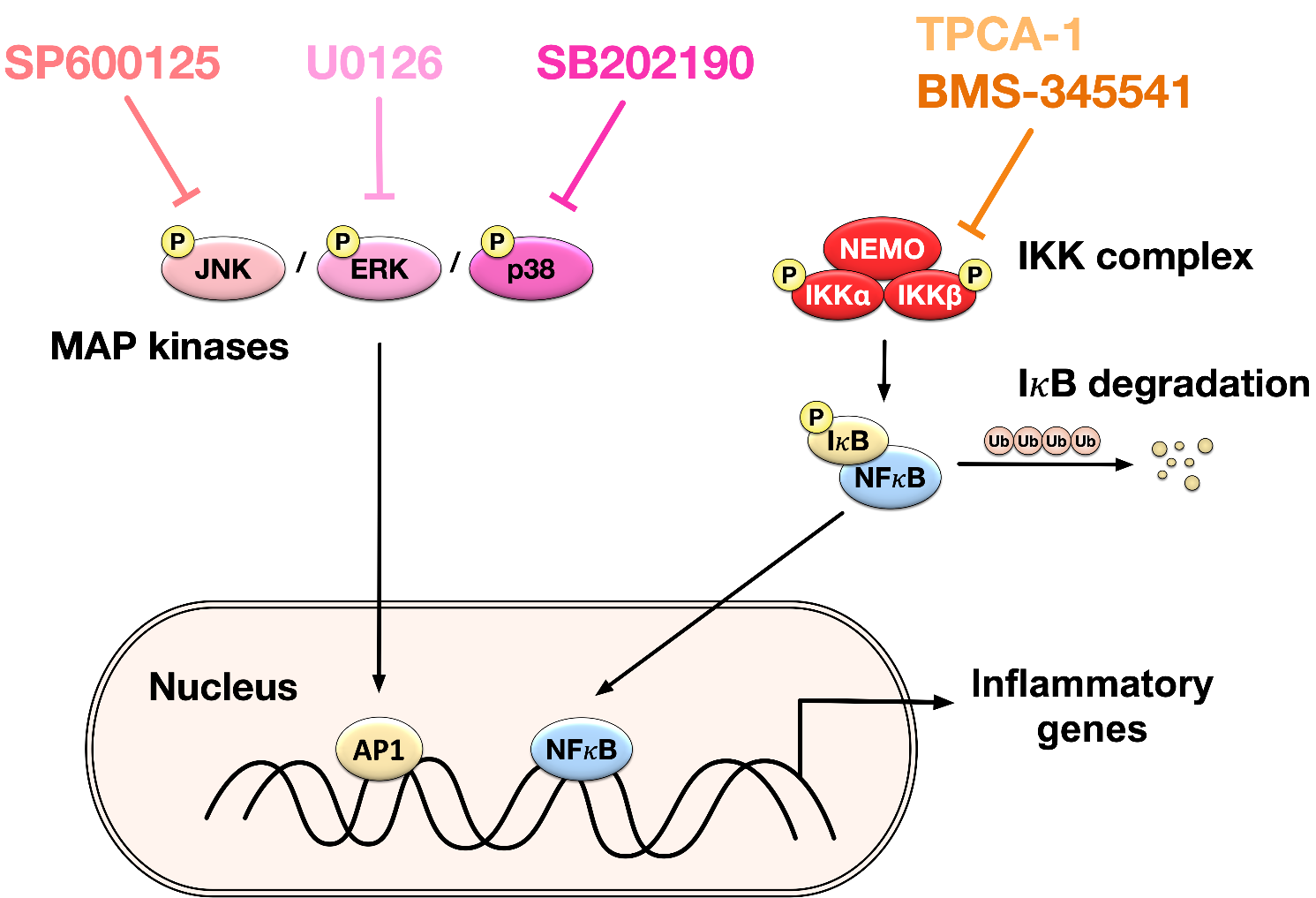
**

**Supplementary Figure 2. Mode of action of the inhibitors used in this study.** Three MAP kinase inhibitors were used in this study: SP600125 as JNK MAP kinase inhibitor, U0126 as ERK MAP kinase inhibitor, and SB202190 as p38 MAP kinase inhibitor. TPCA-1 and BMS-345541 both act as IKK complex inhibitors, blocking the activation of the NFκB pathway.

**
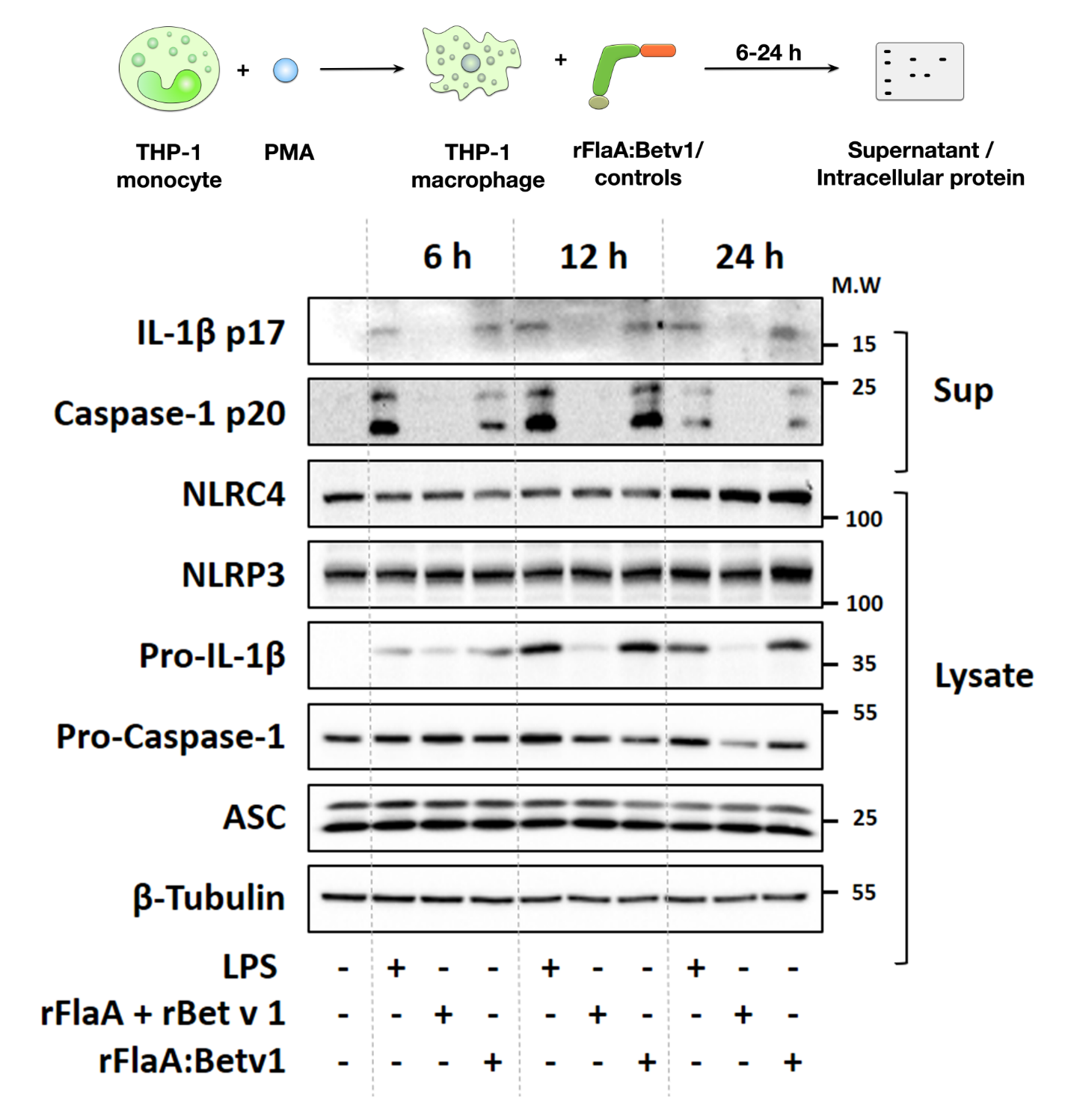
**

**Supplementary Figure 3. rFlaA:Betv1 induces a stronger inflammasome activation in THP-1 macrophages than the mixture of both rFlaA and rBet v 1.** PMA-differentiated THP-1 macrophages were stimulated with either LPS as a positive control or equimolar amounts of either rFlaA + rBet v 1 or rFlaA:Betv1 for 6 to 24 h. Proteins in lysates and supernatants were analyzed by Western Blot, and data are representative results from one experiment out of three experiments that showed similar results.


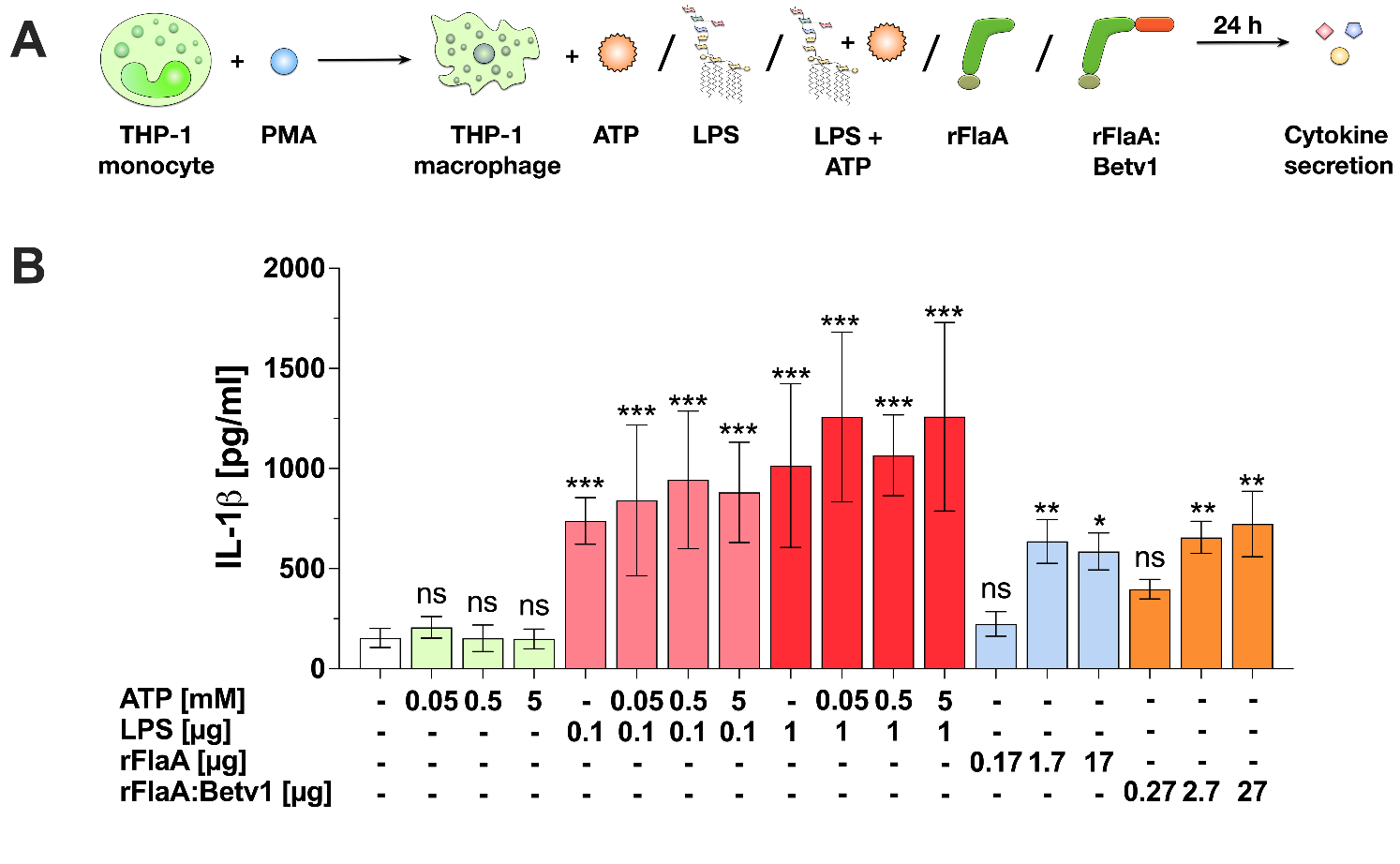


**Supplementary Figure 4.** **Flagellin and the fusion protein rFlaA:Betv1 induce IL-1β secretion in THP-1 cells that is comparable to other well-established inflammasome activators.** PMA-differentiated THP-1 macrophages were stimulated with the indicated concentrations of either ATP, LPS, or LPS + ATP as positive controls to activate the NLRP3 inflammasome or equimolar amounts of either rFlaA or rFlaA:Betv1 for 24 h (**A**). IL-1β secretion in the cells supernatants was analyzed by ELISA (**B**). Data are mean results ± SD from three independent experiments. Statistical significances were calculated in comparison to unstimulated controls and indicated as ns: not significant, *: p-value < 0.05,**: p-value < 0.01, ***: p-value < 0.001.


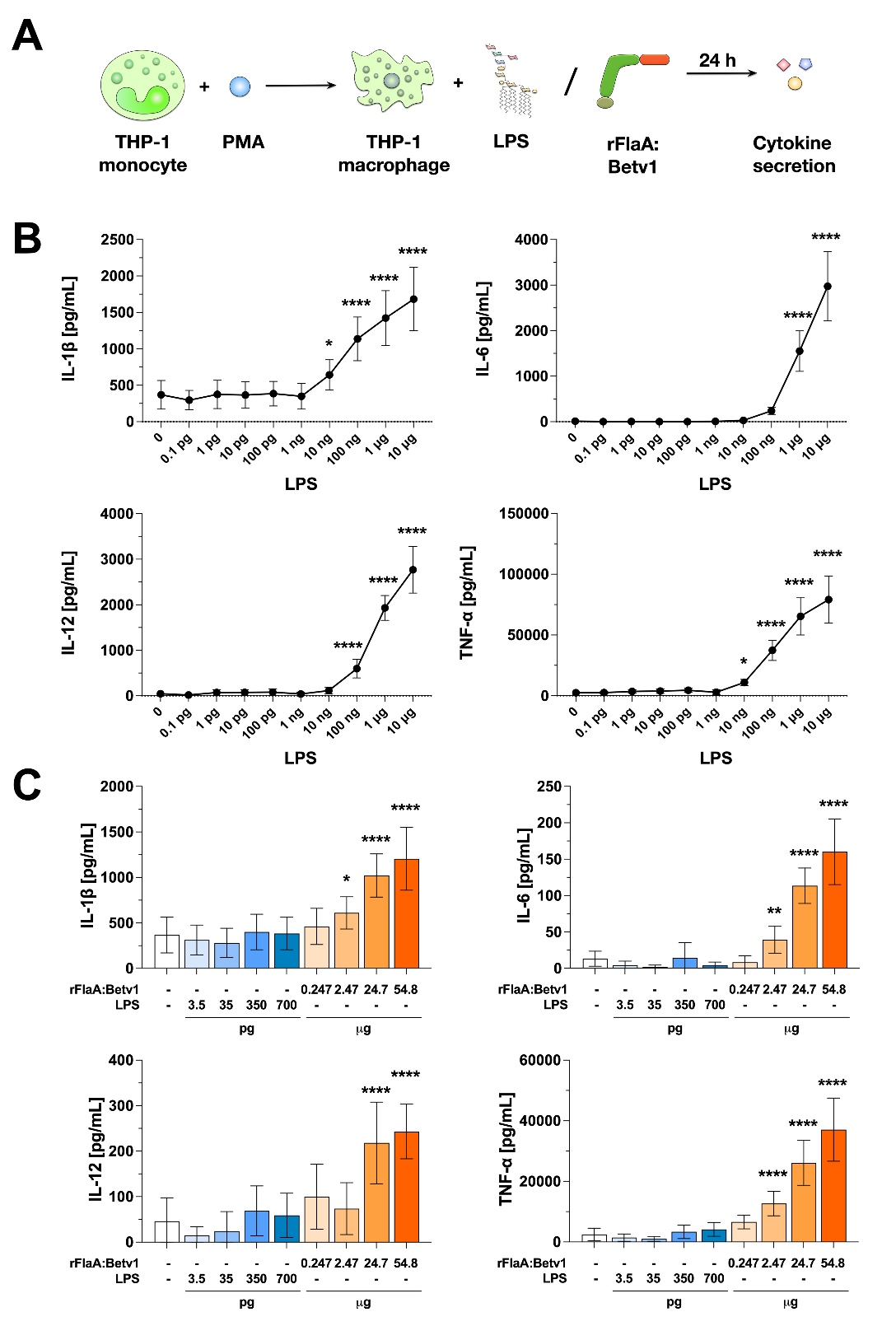


**Supplementary Figure 5.** **Residual endotoxin amounts contained in the used rFlaA:Betv1 preparations are not responsible for the observed cytokine secretion.** Scheme of the performed assays (**A**). PMA-differentiated THP-1 macrophages were stimulated with the indicated amounts of LPS (**B**) or the indicated amounts of rFlaA:Betv1 (orange) and the endotoxin amounts (blue) contained within the respective rFlaA:Betv1 concentrations for 24 h (**C**). Secreted cytokines were analyzed by ELISA. Data are mean results ± SD from three independent experiments. Statistical comparisons were performed between indicated samples and unstimulated control samples, and statistical significances are indicated as *: p-value < 0.05, **: p-value < 0.01, ***: p-value < 0.001**.**


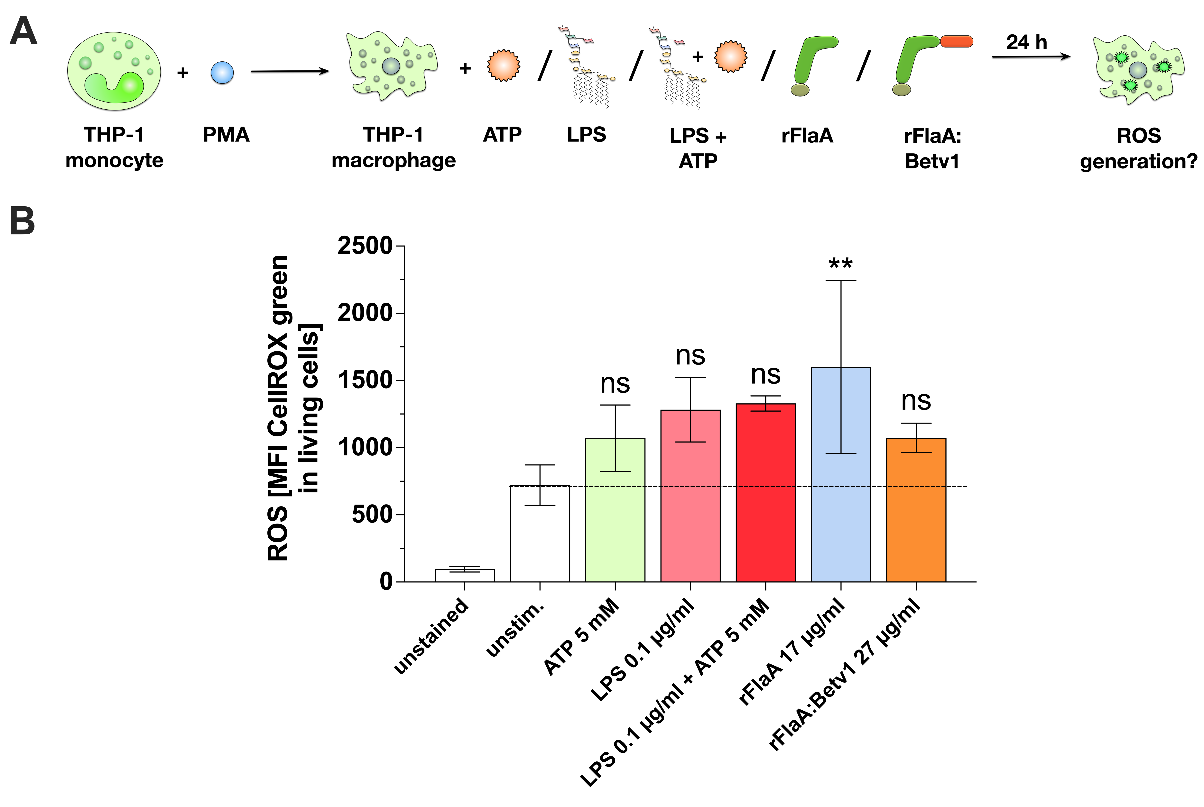


**Supplementary Figure 6.** **Flagellin induces the production of ROS in THP-1 cells.** PMA-differentiated THP-1 macrophages were stimulated with the indicated concentrations of either ATP, LPS, or LPS + ATP as positive controls or equimolar amounts of either rFlaA or rFlaA:Betv1 for 24 h (**A**). Cells were harvested and stained for living cells using the eFlour780 fixable viability dye and ROS production using the CellROX Green reagent. Living cells were analyzed for ROS production by flow cytometry (**B**). Data are mean results ± SD from three independent experiments. Statistical significances are indicated in comparison to unstimulated controls as ns: p-value > 0.05, **: p-value < 0.01.


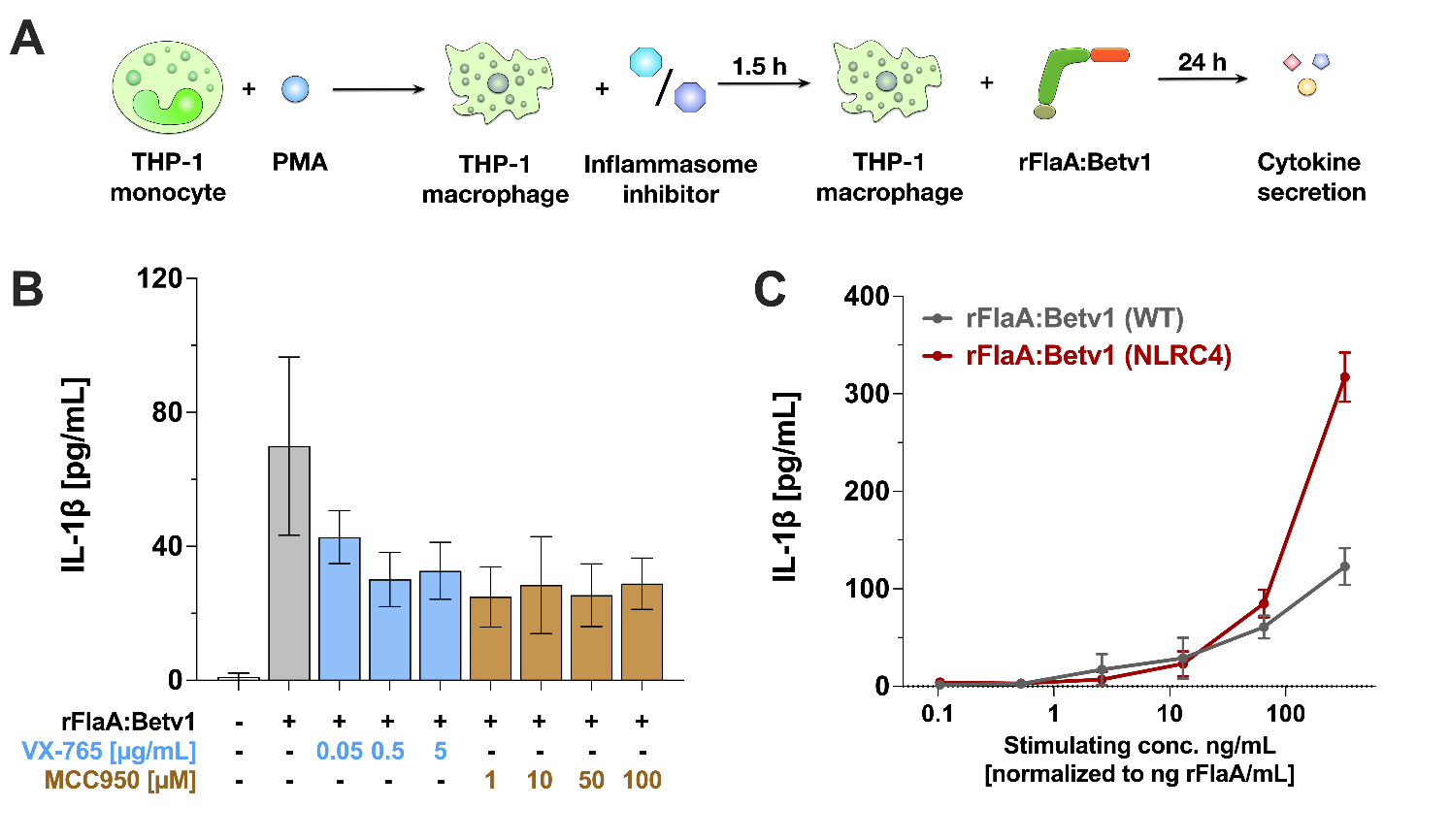


**Supplementary Figure 7.** **Inhibition of inflammasome activation reduces the rFlaA:Betv1-mediated IL-1****β secretion from THP-1 cells while NLRC4 overexpression enhances rFlaA:Betv1-induced IL-1 β secretion.** PMA-differentiated THP-1 macrophages were pre-treated with the indicated inhibitor concentrations for 90 min and subsequently stimulated with 27.4 µg/mL rFlaA:Betv1 for additional 24 h (**A**). Supernatants were collected and examined for the secretion of IL-1β by ELISA (**B**). Wildtype THP-1 (WT) or THP-1 stably overexpressing NLRC4 (NLRC4) were stimulated with the indicated concentrations of rFlaA:Betv1 for 24 h (**C**). Supernatants were collected and examined for the secretion of IL-1β by ELISA. Data are mean results taken from two independent experiments.

**
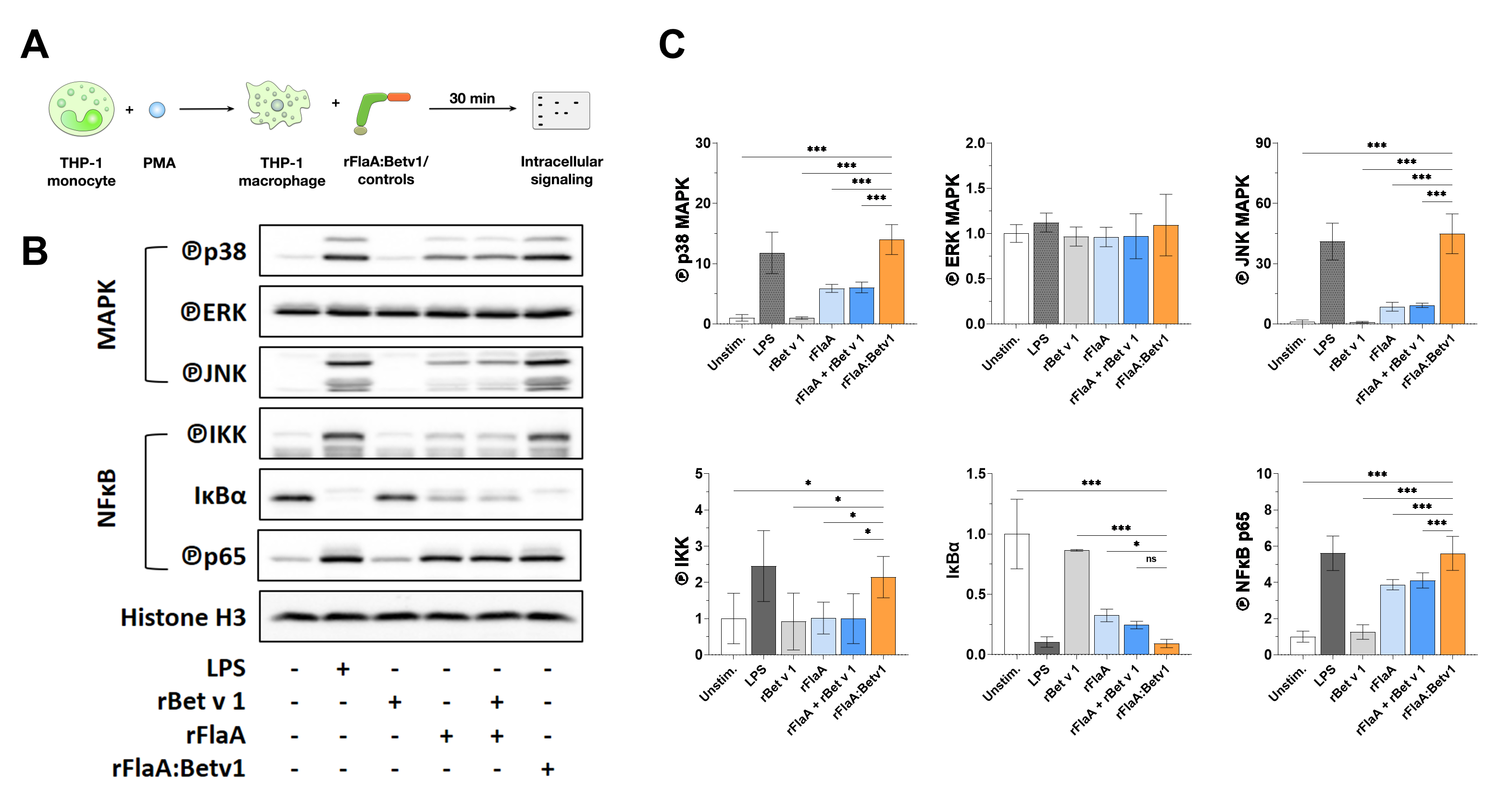
**

**Supplementary Figure 8. Compared to the mixture of both proteins rFlaA:Bev1 induces a stronger activation of MAPK- and NFκB-signaling in THP-1 macrophages.** PMA-differentiated THP-1 macrophages were stimulated with either LPS as a positive control, or equimolar amounts of rFlaA, rBet v 1, rFlaA + rBet v 1, or rFlaA:Betv1 for 30 min (**A**). Cells were lysed and analyzed by Western Blot for the expression levels of the indicated proteins (**B**). The intensities of the Western Blot bands from three independent experiments were analyzed, first normalized to the loading control histone H3 and then again normalized to the unstimulated group, shown as 1 (**C**). Data are either representative (**B**) or mean results of three independent experiments ± SD (**C**). Statistical significances are indicated as ns: p-value > 0.05, *: p-value < 0.05, **: p-value < 0.01, ***: p-value < 0.001.

**
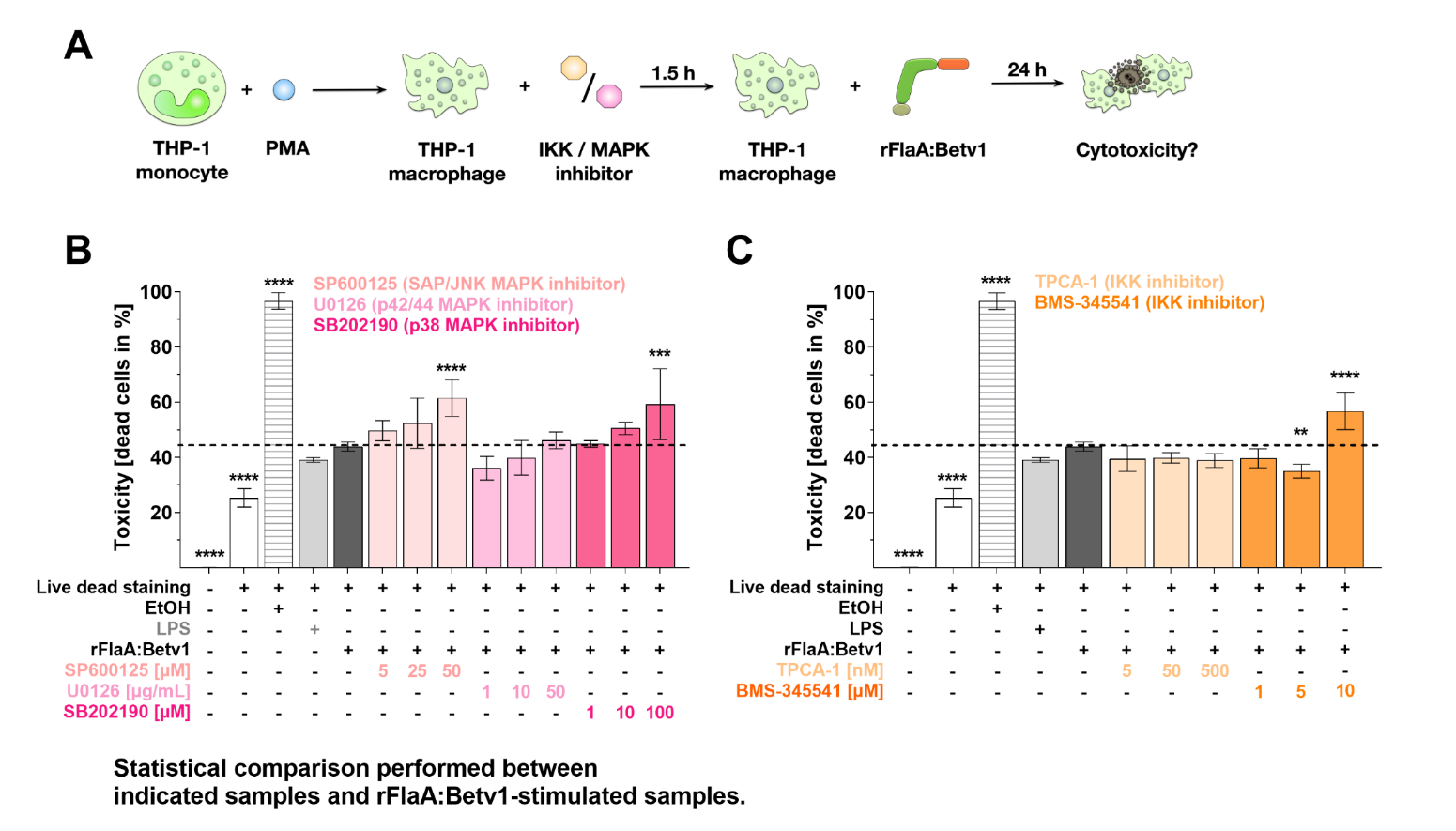
** **Supplementary Figure 9. Cytotoxicity of the used NFKB- and MAPK-inhibitors on THP-1 macrophages.** PMA-differentiated THP-1 macrophages were pre-treated with the indicated concentrations of inhibitors for 90 min, followed by stimulation with 27.4 μg/mL of rFlaA:Betv1 for additional 24 h. Cells were harvested, stained for dead cells using fixable viability dye, and the percentage of dead cells was analyzed by flow cytometry. Cells killed by incubation with 70% ethanol for 5 min were used as positive controls. In subsequent stimulation experiments, inhibitor concentrations that showed toxic effects were excluded. Data are the mean results of three independent experiments ± SD. Statistical comparisons between the indicated samples and rFlaA:Betv1-stimulated samples were performed and statistical significance was shown as *: p-value < 0.05, **: p-value < 0.01, ***: p-value < 0.001.
